# Role of TBATB in nano indium oxide catalyzed C-S bond formation

**DOI:** 10.1038/srep13873

**Published:** 2015-09-29

**Authors:** Prasanta Gogoi, Sukanya Hazarika, Pranjit Barman

**Affiliations:** 1 Department of Chemistry, National Institute of Technology, Silchar 788010, Assam, India

## Abstract

Nano sized indium oxide is found to be an efficient catalyst for the conversion of thiols to sulfides using Na_2_CO_3_ as base and TBATB as reagent in DMSO at 110 °C. Here *in situ* generation of bromo intermediate by TBATB takes place through indium surface. A variety of aryl sulfides can be synthesized in excellent yields from less reactive chlorides, boronic acids and thiols.

The applications of metal-nanoparticle catalysis in organic synthesis has received considerable interest in recent years^1^. One of the important applications is toward the C-S bond formation has increased tremendously because of its high efficiency and compatibility with eco-friendly reaction media^2^. Transition metal-catalyzed cross-coupling is one of the most commonly used reactions in the construction of C-S bonds, in which the formation of diaryl sulfide motifs is particularly valuable due to its significance in biologically active molecules and chemical materials^3^. During the past decades, various Cu 4,5, Ni 6, Pd 7,8, Co 9, and Fe 10,11 catalytic systems have been developed that allow aryl halides and aryl boronic acids that couple with arylthiols under mild reaction conditions. In the recent decades, however, indium-based^12^ catalytic systems have attracted considerable attention due to their high reactivity, low cost and lower toxicity. The indium-catalyzed C-S cross coupling of organyl halides with dichalcogenides was described in 2009^13^,^14^. Moreover, Reddy and co-worker^15^ reported nano-In_2_O_3_ catalyzed C-S cross-coupling of aryl halides with aromatic/alkyl thiols but this protocol reported a longer reaction time. Nevertheless, to the best of our knowledge, their application as catalysts for the C-S cross-coupling reaction of thiol with arylboronic acid has yet not been discovered. We report herein a facile synthesis of aryl thioethers employing nano indium oxide as catalyst in several transformations (Fig. 1). The catalyst efficiency can be increased by using TBATB (tetrabutylammonium tribromide).

## Results

**Figure 2:**
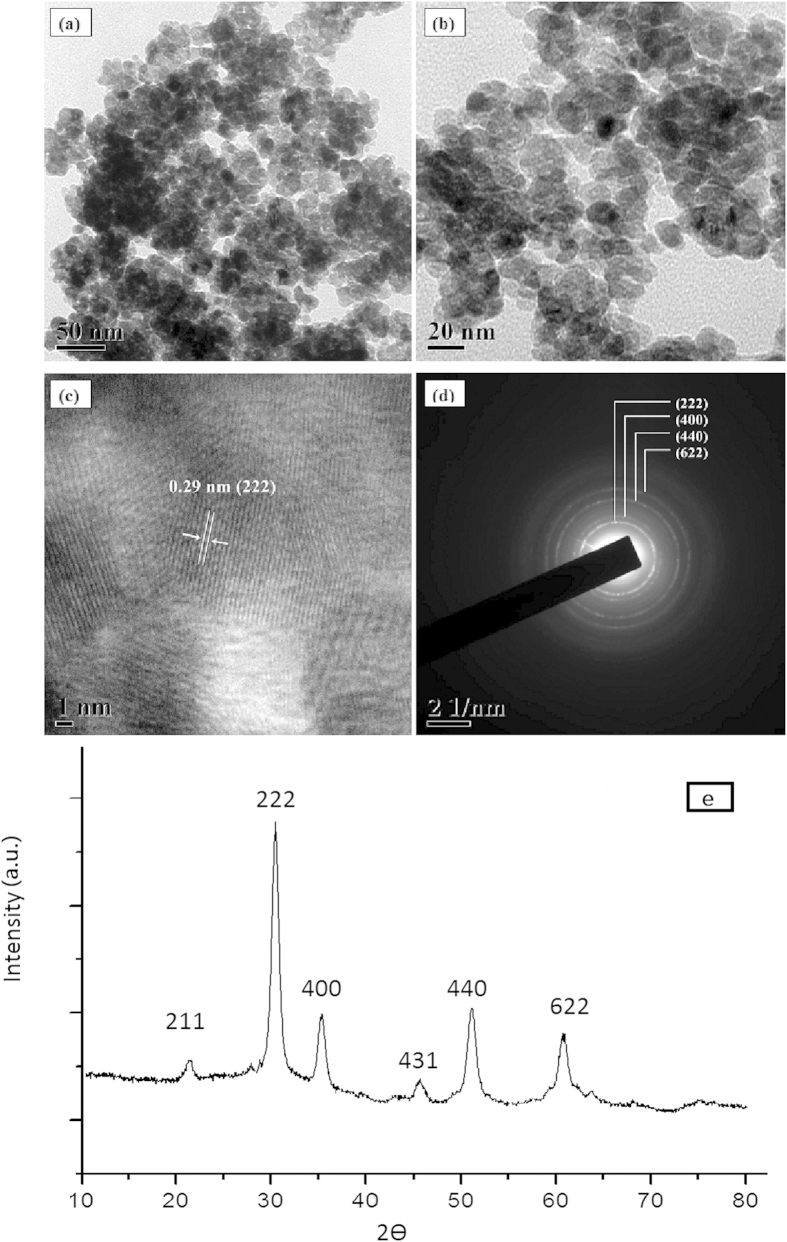
TEM-images of In_2_O_3_ nanoparticles at **(a,b)** lower and **(c)** higher magnification **(d)** SAED pattern **(e)** powder XRD.

**Figure 3:**
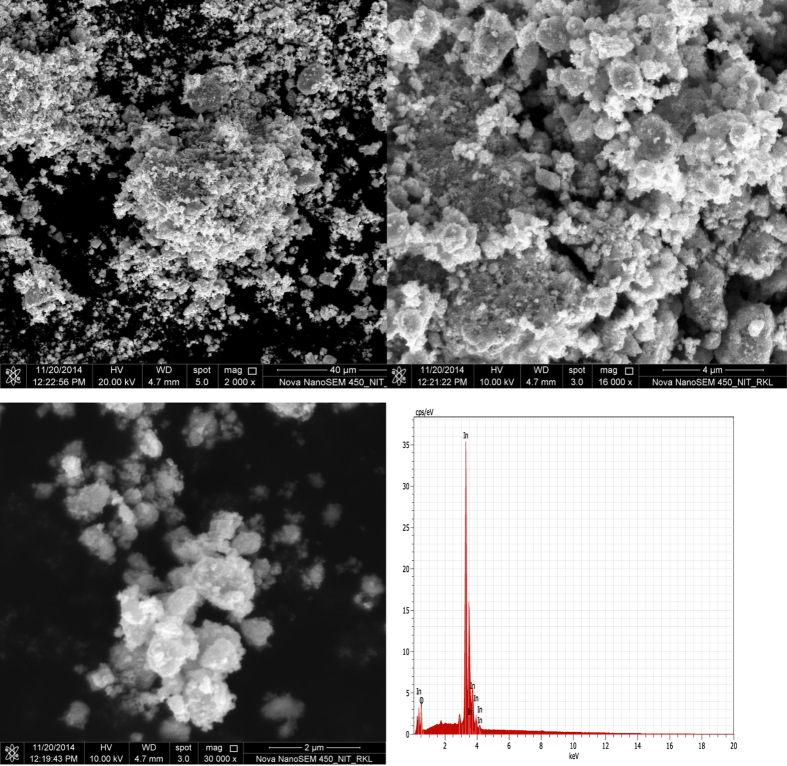
SEM-images of In_2_O_3_ nanoparticles at **(a,b)** lower and **(c)** higher magnification **(d)** Energy dispersive X-ray spectroscopy (EDS).

Indium oxide nanoparticles (NPs) could be synthesized following a reported method^16^ with minor modifications. In a typical procedure, 0.10 mol ratio of In(NO_3_)_2_9H_2_O were added to 20 ml distilled water in a three necked round-bottom flask and stirred at 100 °C for 30 min. 0.12 M NaOH solution were added dropwise untill white precipitate was obtained (pH = 9). The obtained precipitate was centrifuged and washed three times with double distilled water. The final white product was calcined at 400 °C. After calcination, the colour of the powder turned from white to light yellow indicating that the resultant product is In_2_O_3_. To investigate the composition and topography of the final calcination product, powder XRD, scanning electron microscopy (SEM) image and EDS were carried out. Figures (2) and (3) shows the typical transmission electron microscopy (TEM) image and scanning electron microscopy (SEM) image of indium oxide nanoparticles, where the sizes are estimated to be 10–20 nm.

**Figure 4:**
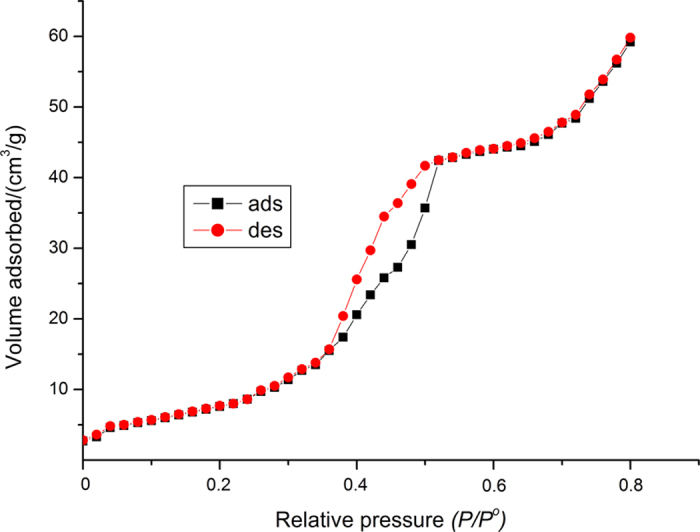
N_2_ adsorption-desorption isotherm of indium oxide nanoparticles.

The N_2_ adsorption and desorption isotherm for nano indium oxide is type IV (Fig. 4). The surface area and average pore diameter of the prepared nano-In_2_O_3_ were 66.9 m^2^ g^−1^ and 7.7 nm, respectively.

To optimize the reaction conditions a series of experiments were performed under varying reaction parameters, such as solvent, time, temperature and base for a representative C-S cross coupling reaction. In the preliminary phase of optimization, thiophenol and allyl chloride was chosen as a standard substrate and the trials were performed with different catalysts, ligands, and solvents at different temperature (Table 1). Nano-In_2_O_3_ was found to be the best choice among the various catalysts (Table 1, entry 1). The results shown that 2 equiv Na_2_CO_3_ and 1.5 equiv TBATB with 2 mol% nano-In_2_O_3_ were the best concentrations for the transformation to occur within a reasonable time period. When 5 moI% of catalyst was used, no appreciable change with respect to yield was observed (Table 1, entry 3). Nevertheless, other catalyst (Co_2_CO_3_) gave low yield. The coupling reaction was investigated in various solvents such as CH_2_Cl_2_, DMSO, CH_3_CN and CH_3_COOH. DMSO was found to be the best solvent.

**Figure 5:**
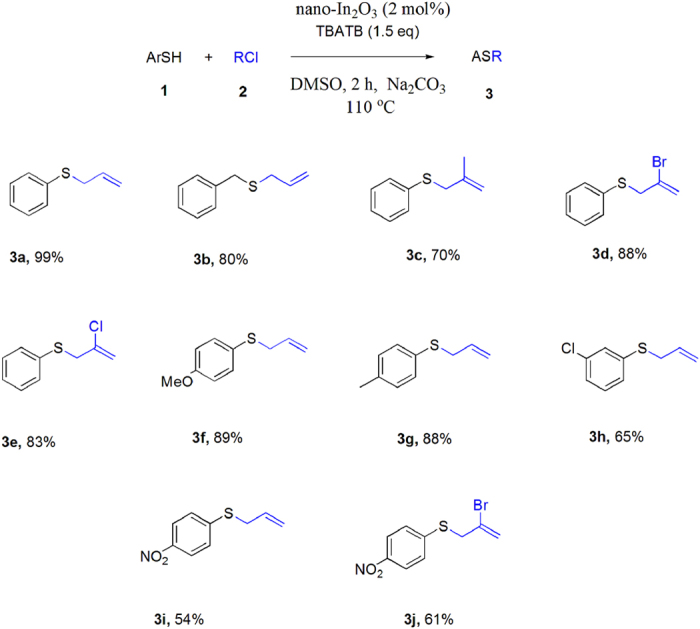
Synthesis of Aryl-allyl sulfide: Reactions were carried out with allyl chloride (0.2 mmol) Arylthiol (0.2 mmol), nano-In_2_O_3_ (2 mol%), TBATB (1.5 equiv), Na_2_CO_3_ (2.0 equiv) in DMSO (2 mL) for 2 h.

Having optimized conditions (Table 1 entry 1) for C-S reaction, we continued our pursuit with a variety of aromatic and aliphatic substrates (Fig. 5). As shown in Fig. 5, aryl thiols bearing electron-donating groups such as methoxy, methyl groups (**3f-g**) efficiently couple with allyl chlorides to yield the corresponding arylsulfide in excellent yields (88–89%). However, arylthiols with electron withdrawing groups gave the corresponding arylsulfides in lower yields (**3i-j**) and benzylthiols gave moderate to good yields (**3b**).

**Figure 6:**
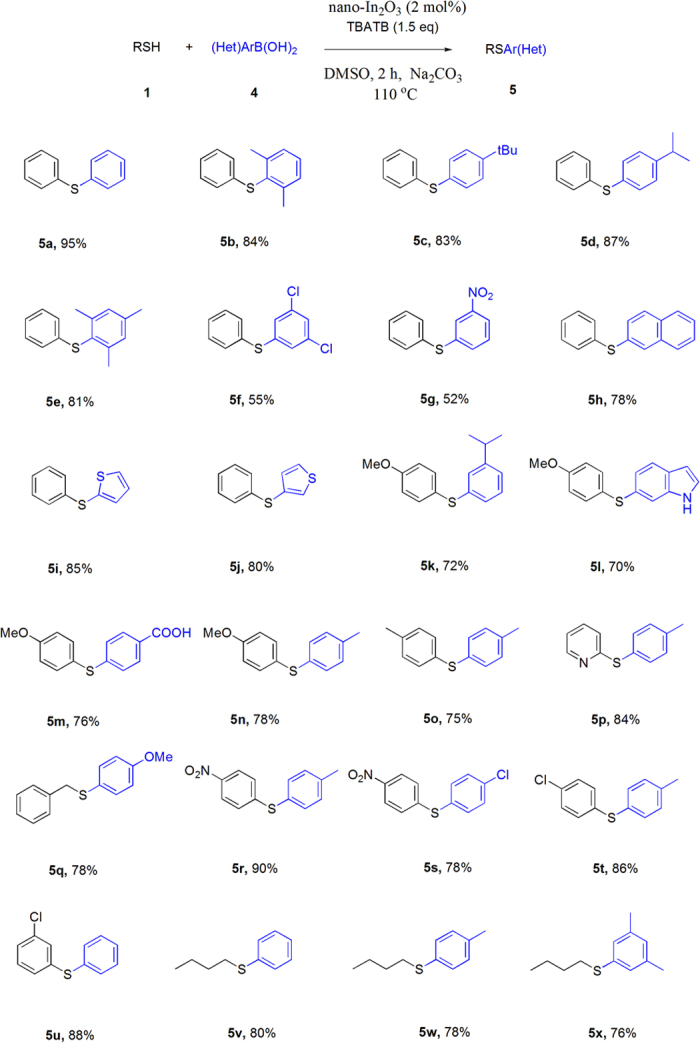
Coupling of thiols with arylboronic acids: Reactions were carried out with arylboronic acid (0.2 mmol), thiol (0.2 mmol), nano-In_2_O_3_ (2 mol%), TBATB (1.5 equiv), Na_2_CO_3_ (2.0 equiv) in DMSO (2 mL) for 2 h.

Having same standard reaction conditions, we explored the scope of thiols with arylboronic acids (Fig. 6). A broad range of functional groups, such as ether, halides, ester, etc. were tolerated under the standard reaction condition to provide the expected products, **5a-x** in good to excellent yields. Aryl thiols containing electron withdrawing and electron donating group were coupled to furnish the corresponding diarylsulfides in good to excellent yields. It is noteworthy that our protocol could also be extended to alkyl thiols giving good yields. Quite significantly, a wide range of boronic acids that incorporate electron donating groups (**5b-e**) and electron-withdrawing groups at the ortho, meta, and para positions are readily tolerated. However, low yields of respective diarylsulfides were obtained with 3-nitro-phenylboronic acid, 3,5-dichlorophenyl boronic acid and 4-chlorophenylboronic acid (**5f-g, 5s**).

**Figure 7:**
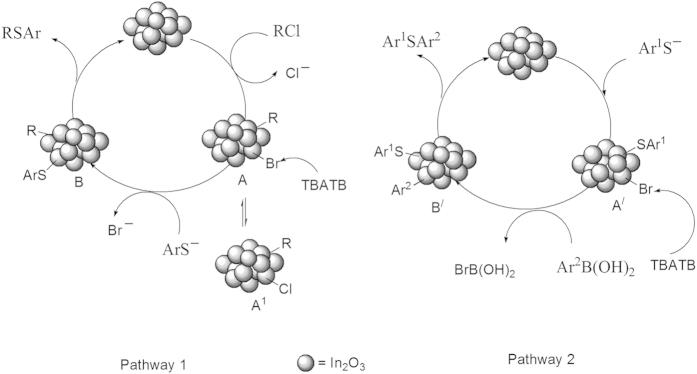
Plausible mechanism for C-S bond formation

On the basis of previous literature reports^17^, the proposed reaction pathway for these reactions is based on oxidative addition followed by reductive elimination (Fig. 7). The consequent oxidative addition (pathway1) of In(nano) with allyl chloride may provide intermediate RIn(nano)Br (A) or RIn(nano)Cl A^1^. The product yield increased suddenly on addition of TBATB and might be it follows the formation of intermediate (A) largely rather than A^1^. Then in presence of base thiol react with A can give intermediate B, which undergoes a reductive elimination to provide the target product and to regenerate the catalyst In(nano).

In pathway 2, ArSH on oxidative addition to form intermediate A^/^ and in presence of base arylboronic acid react with A^/^ to form intermediate B^/^ which undergo reductive elimination to form the product. The main significance of this work that In(nano) provides large surface area for which the Br^-^ can easily form intermediate A or A^/^.

**Figure 8:**
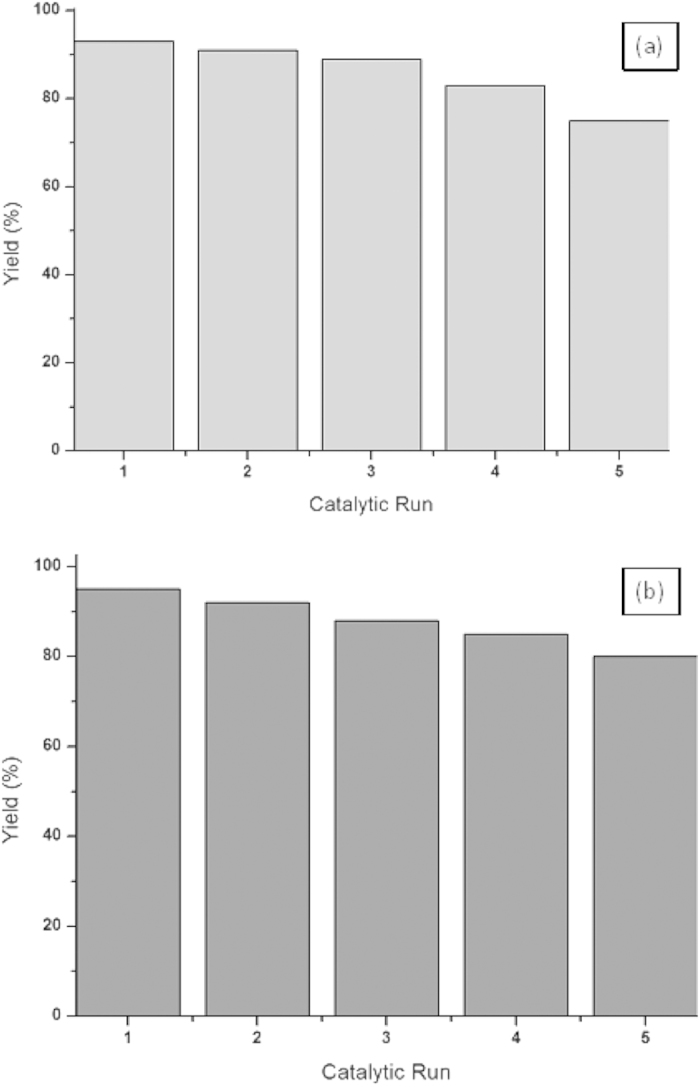
Recovery of In_2_O_3_ nps: (**a**) Reactions were carried out with allyl chloride (0.2 mmol) phenylthiol (0.2 mmol), nano-In_2_O_3_ (2 mol%), TBATB (1.5 equiv), Na_2_CO_3_ (2.0 equiv) in DMSO (2 mL) for 2 h. **(b)** Reactions were carried out with phenyIboronic acid (0.2 mmol), phenyIthiol (0.2 mmol), nano-In_2_O_3_ (2 mol%), TBATB (1.5 equiv), Na_2_CO_3_ (2.0 equiv) in DMSO (2 mL) for 2 h.

After completion of reaction the catalyst was recovered by centrifugation and reused for the fresh reaction of aryl thiol with allyl chloride/boronic acid and no loss of activity was observed (Fig. 8).

We have reported a general synthetic protocol for the C-S bond, using nano-indium-catalyst. In this protocol, we recommend the use of 2 moI% indium-catalyst, TBATB (1.5 equiv) Na_2_CO_3_ (2 equiv), and DMSO as the solvent. We found the main advantages of this protocol were low toxicity of the catalyst, good turnover and high yield of products.

## Methods

### General Information

All solvents and chemicals were purchased commercially and used without further purification. Melting points were recorded on an electrothermal digital melting point apparatus and were uncorrected. Column chromatography was generally performed on silica gel (230–400 mesh) and reactions were monitored by thin layer chromatography (TLC) using UV light (254 nm) to visualize the course of reactions. ^1^H and ^13^C Nuclear Magnetic Resonance spectra of pure compounds were acquired at 400 and 100 MHz respectively. All NMR samples were recorded in deuterated chloroform. Chemical shifts (ppm) were recorded with tetramethylsilane (TMS) as the internal reference standard. Elemental analyses were performed on a Flash 2000 Thermo Scientific instrument at NIT Silchar. The TEM and SEM characterization were carried out at model no. CM-12 Philips TEM (IIT Kharagpur) and FEI Nova nano-SEM-450 (NIT Rourkela) respectively.

### Allyl(phenyl)sulfane (3a)^18^

To a 10 mL glass tube, In_2_O_3_ nanoparticles (1.1 mg, 0.004 mmol), TBATB (144 mg, 0.3 mmol), Na_2_CO_3_ (42 mg, 0.4 mmol), thiophenol (22 mg, 0.2 mmol) and allyl chloride (15.2 mg, 0.2 mmol) were dissolved in DMSO (2 mL) under N_2_ atmosphere. The tube was sealed and the mixture was stirred at the corresponding temperature for 2 h. The reaction mixture was filtered and the solvent evaporated in vacuo to give the crude product, which was purified by column chromatography (hexane/ethyl acetate = 25/1) to give the title compound **3a** (149 mg, 99%) as a light yellow liquid; [Found: C, 71.98; H, 6.49; C_9_H_10_S. requires C, 71.95; H, 6.71%]; R*f* = 0.4 (hexane/ethyl acetate = 20/1). ^1^H NMR (400 MHz, CDCl_3_): δ 7.39–7.28 (m, 5H), 5.94 (m, 1H), 5.28 (dd, *J* *=* 1.3 Hz, *J* *=* 16.4 Hz, 1H), 5.13 (dd, *J* *=* 1.2 Hz, *J* *=* 10.1 Hz, 1H),3.68 (d, *J* *=* 6.8 Hz, 2H) ^13^C NMR (100 MHz, CDCl_3_): δ 134.9, 133.9, 130.7, 129.8, 126.9, 118.9, 37.4.

### (2-methylallyl)(phenyl)sulfane (3c)^19^

To a 10 mL glass tube, In_2_O_3_ nanoparticles (1.1 mg, 0.004 mmol), TBATB (144 mg, 0.3 mmol), Na_2_CO_3_ (42 mg, 0.4 mmol), thiophenol (22 mg, 0.2 mmol) and 2-chloro-2-methyl-propene (18.2 mg, 0.2 mmol) were dissolved in DMSO (2 mL) under N_2_ atmosphere. The tube was sealed and the mixture was stirred at the corresponding temperature for 2 h. The reaction mixture was filtered and the solvent evaporated in vacuo to give the crude product, which was purified by column chromatography (hexane/ethyl acetate = 10/1) to give the title compound **3c** (114.8 mg, 70%) as a colourless liquid; [Found: C, 72.98; H, 7.39; C_10_H_12_S requires C, 73.12; H, 7.36%]; R*f* = 0.4 (hexane/ethyl acetate = 10/1). ^1^H NMR (400 MHz, CDCl_3_): δ 7.44–7.33 (m, 5H), 5.16 (d, *J* *=* 1.2 Hz, 1H), 4.89 (d, *J* *=* 1.1 Hz, 1H), 3.65 (s, 2H), 1.87 (s, 3H). ^13^C NMR (100 MHz, CDCl_3_): δ 142.4, 135.3, 131.2, 129.1, 128.2, 112.5, 42.9, 21.7.

### (2-bromoallyl)(phenyl)sulfane (3d)^20^

To a 10 mL glass tube, In_2_O_3_ nanoparticles (1.1 mg, 0.004 mmol), TBATB (144 mg, 0.3 mmol), Na_2_CO_3_ (42 mg, 0.4 mmol), thiophenol (22 mg, 0.2 mmol) and 2-bromo-allyl bromide (39.6 mg, 0.2 mmol) were dissolved in DMSO (2 mL) under N_2_ atmosphere. The tube was sealed and the mixture was stirred at the corresponding temperature for 2 h. The reaction mixture was filtered and the solvent evaporated in vacuo to give the crude product, which was purified by column chromatography (hexane/ethyl acetate = 15/1) to give the title compound **3d** (201 mg, 88%) as a colourless liquid; [Found: C, 47.16; H, 3.93; S, 14.04; C_9_H_9_BrS requires C, 47.18; H, 3.96; S, 13.99%]; R*f* = 0.4 (hexane/ethyl acetate = 15/1). ^1^H NMR (400 MHz, CDCl_3_): δ 7.53–7.30 (m, 5H), 5.89 (d, *J* *=* 1.8 Hz, 1H), 5.50 (d, *J* *=* 1.7 Hz, 1H), 4.01 (m, 2H). ^13^C NMR (100 MHz, CDCl_3_): δ 135.2,131.1, 129.4, 129.2, 127.5, 119.6, 45.2.

### (2-chloroallyl)(phenyl)sulfane (3e)

To a 10 mL glass tube, In_2_O_3_ nanoparticles (1.1 mg, 0.004 mmol), TBATB (144 mg, 0.3 mmol), Na_2_CO_3_ (42 mg, 0.4 mmol), thiophenol (22 mg, 0.2 mmol) and 2-chloro-allyl chloride (22 mg, 0.2 mmol) were dissolved in DMSO (2 mL) under N_2_ atmosphere. The tube was sealed and the mixture was stirred at the corresponding temperature for 2 h. The reaction mixture was filtered and the solvent evaporated in vacuo to give the crude product, which was purified by column chromatography (hexane/ethyl acetate = 20/1) to give the title compound **3e** (153 mg, 83%) as a colourless liquid; [Found: C, 58.58; H, 4.80; C_9_H_9_ClS requires C, 58.53; H, 4.91%]; R*f* = 0.4 (hexane/ethyl acetate = 20/1). ^1^H NMR (400 MHz, CDCl_3_): δ 7.94–7.86 (m, 5H), 5.77–5.64 (m, 2H), 3.88 (d, *J* *=* 6.3 Hz, 2H). ^13^C NMR (100 MHz, CDCl_3_): δ 140.8, 137.7, 133.9, 128.4, 126.2, 119.7, 44.1.

### Allyl(*p-*tolyl)sulfane (3g)^21^

To a 10 mL glass tube, In_2_O_3_ nanoparticles (1.1 mg, 0.004 mmol), TBATB (144 mg, 0.3 mmol), Na_2_CO_3_ (42 mg, 0.4 mmol), 4-methylthiophenol (24.8 mg, 0.2 mmol) and allyl chloride (15.2 mg, 0.2 mmol) were dissolved in DMSO (2 mL) under N_2_ atmosphere. The tube was sealed and the mixture was stirred at the corresponding temperature for 2 h. The reaction mixture was filtered and the solvent evaporated in vacuo to give the crude product, which was purified by column chromatography (hexane/ethyl acetate = 15/1) to give the title compound **3g** (144 mg, 88%) as a colourless liquid; [Found: C, 73.13; H, 7.31. C_10_H_12_S. requires C, 73.12; H, 7.36%]; R*f* = 0.5 (hexane/ethyl acetate = 10/1). ^1^H NMR (400 MHz, CDCl_3_): δ 7.38 (d, *J* *=* 8.1 Hz, 2H), 7.03 (d, *J* *=* 7.9 Hz, 2H), 5. 74–5.55 (m, 1H), 5.16–5.06 (m, 2H), 3.58 (d, *J* = 7.6 Hz, 2H), 2.35 (s, 3H).^13^C NMR (100 MHz, CDCl_3_): δ 138.7, 134.2, 132.8,131.6, 129.5, 117.9, 37.2, 20.1

### allyl(3-chlorophenyl)sulfane (3 h)

To a 10 mL glass tube, In_2_O_3_ nanoparticles (1.1 mg, 0.004 mmol), TBATB (144 mg, 0.3 mmol), Na_2_CO_3_ (42 mg, 0.4 mmol), 3-chlorothiophenol (28.8 mg, 0.2 mmol) and allyl chloride (15.2 mg, 0.2 mmol) were dissolved in DMSO (2 mL) under N_2_ atmosphere. The tube was sealed and the mixture was stirred at the corresponding temperature for 2 h. The reaction mixture was filtered and the solvent evaporated in vacuo to give the crude product, which was purified by column chromatography (hexane/ethyl acetate = 15/1) to give the title compound **3h** (105 mg, 65%) as a yellow liquid; [Found: C, 58.58; H, 4.94; C_9_H_9_ClS requires C, 58.53; H, 4.91%]; R*f* = 0.5 (hexane/ethyl acetate = 15/1). ^1^H NMR (400 MHz, CDCl_3_): δ 7.38–7.29 (m, 4H), 5.86–5.75 (m, 1H), 5.27 (dd, *J* *=* 1.1 Hz, *J* *=* 16.1 Hz, 1H), 5.17 (dd, *J* *=* 1.3 Hz, *J* *=* 10.2 Hz, 1H), 3.59 (d, *J* *=* 6.1 Hz, 2H). ^13^C NMR (100 MHz, CDCl_3_): δ 137.7, 134.2, 132.5, 128.9, 127.9, 125.6, 124.3, 117.7, 35.9.

### Allyl(4-nitrophenyl)sulfane (3i)^22^

To a 10 mL glass tube, In_2_O_3_ nanoparticles (1.1 mg, 0.004 mmol), TBATB (144 mg, 0.3 mmol), Na_2_CO_3_ (42 mg, 0.4 mmol), 4-nitrothiophenol (31 mg, 0.2 mmol) and allyl chloride (15.2 mg, 0.2 mmol) were dissolved in DMSO (2 mL) under N_2_ atmosphere. The tube was sealed and the mixture was stirred at the corresponding temperature for 2 h. The reaction mixture was filtered and the solvent evaporated in vacuo to give the crude product, which was purified by column chromatography (hexane/ethyl acetate = 15/1) to give the title compound **3i** (105 mg, 54%) as a yellow solid, mp 39–41 °C; [Found: C, 55.31; H, 4.66; N, 6.99; C_9_H_9_NO_2_S requires C, 55.37; H, 4.65; N, 7.17%]; R*f* = 0.6 (hexane/ethyl acetate = 15/1). ^1^H NMR (400 MHz, CDCl_3_): δ 8.45 (d, *J* *=* 8.1 Hz, 2H), 7.38 (d, *J* *=* 8.6 Hz, 2H), 5.89–5.83 (m, 1H), 5.36 (dd, *J* *=* 1.1 Hz, *J* *=* 16.3 Hz, 1H), 5.27 (dd, *J* *=* 1.4 Hz, *J* *=* 10.1 Hz, 1H), 3.76 (d, *J* *=* 6.3 Hz, 2H). ^13^C NMR (100 MHz, CDCl_3_): δ 147.8, 146.7, 133.9, 127.8, 124.8, 119.4, 35.2.

### (2-bromoallyl)(4-nitrophenyl)sulfane (3j)

To a 10 mL glass tube, In_2_O_3_ nanoparticles (1.1 mg, 0.004 mmol), TBATB (144 mg, 0.3 mmol), Na_2_CO_3_ (42 mg, 0.4 mmol), 4-nitrothiophenol (31 mg, 0.2 mmol) and 2-bromo-allyl bromide (39.6 mg, 0.2 mmol) were dissolved in DMSO (2 mL) under N_2_ atmosphere. The tube was sealed and the mixture was stirred at the corresponding temperature for 2 h. The reaction mixture was filtered and the solvent evaporated in vacuo to give the crude product, which was purified by column chromatography (hexane/ethyl acetate = 10/1) to give the title compound **3j** (167 mg, 61%) as a colourless liquid; [Found: C, 39.49; H, 2.99; N, 5.01; C_9_H_8_BrNO_2_S requires C, 39.43; H, 2.94; N, 5.11%]; R*f* = 0.4 (hexane/ethyl acetate = 10/1). ^1^H NMR (400 MHz, CDCl_3_): δ 8.20 (d, *J* *=* 9.1 Hz, 2H), 7.36 (d, *J* *=* 8.2 Hz, 2H), 5.77 (d, *J* *=* 2.9 Hz, 1H), 5.47 (d, *J* *=* 2.6 Hz, 1H), 4.01 (s, 2H). ^13^C NMR (100 MHz, CDCl_3_): δ 143.1, 139.2, 125.7, 124.9, 121.1, 44.8.

### Diphenyl sulfide (5a)^23^

To a 10 mL glass tube, In_2_O_3_ nanoparticles (1.1 mg, 0.004 mmol), TBATB (144 mg, 0.3 mmol), Na_2_CO_3_ (42 mg, 0.4 mmol), thiophenol (22 mg, 0.2 mmol) and phenylboronic acid (24.4 mg, 0.2 mmol) were dissolved in DMSO (2 mL) under N_2_ atmosphere. The tube was sealed and the mixture was stirred at 110 °C. The reaction mixture was filtered and the solvent evaporated in vacuo to give the crude product, which was purified by column chromatography (hexane/ethyl acetate = 15/1) to give the title compound **5a** (177 mg, 95%) as a colourless liquid; [Found: 77.55; H, 5.47; C_12_H_10_S requires C, 77.37; H, 5.41%]; R*f* = 0.4 (hexane/ethyl acetate = 15/1). ^1^H NMR (400 MHz, CDCl_3_): δ 7.40 – 7.27 (m, 10H). ^13^C NMR (100 MHz, CDCl_3_): δ 135.5, 131.2, 129.2, 127.7.

### 2, 6-Dimethylphenyl phenyl sulfide (5b)^24^

To a 10 mL glass tube, In_2_O_3_ nanoparticles (1.1 mg, 0.004 mmol), TBATB (144 mg, 0.3 mmol), Na_2_CO_3_ (42 mg, 0.4 mmol), thiophenol (22 mg, 0.2 mmol) and 2,6-dimethyl-phenylboronic acid (30 mg, 0.2 mmol) were dissolved in DMSO (2 mL) under N_2_ atmosphere. The tube was sealed and the mixture was stirred at 110 °C. The reaction mixture was filtered and the solvent evaporated in vacuo to give the crude product, which was purified by column chromatography (hexane/ethyl acetate = 20/1) to give the title compound **5b** (180 mg, 84%) as a colourless liquid; [Found: C, 78.65; H, 6.54; C14H14S requires C, 78.45; H, 6.58%]; R*f* = 0.4 (hexane/ethyl acetate = 20/1). ^1^H NMR (400 MHz, CDCl_3_): δ 7.28–7.22 (m, 5H), 7.10– 6.96 (m, 3H), 2.46 (s, 6H). ^13^C NMR (100 MHz, CDCl_3_): δ 144.1, 138.2, 130.7, 129.4, 128.4, 128.0, 125.3, 124.0, 21.4.

### 4-*tert*-Butylphenyl phenyl sulfide (5c)

To a 10 mL glass tube, In_2_O_3_ nanoparticles (1.1 mg, 0.004 mmol), TBATB (144 mg, 0.3 mmol), Na_2_CO_3_ (42 mg, 0.4 mmol), thiophenol (22 mg, 0.2 mmol) and 4-*tert*-butylphenylboronic acid (35.6 mg, 0.2 mmol were dissolved in DMSO (2 mL) under N_2_ atmosphere. The tube was sealed and the mixture was stirred at 110 °C. The reaction mixture was filtered and the solvent evaporated in vacuo to give the crude product, which was purified by column chromatography (hexane/ethyl acetate = 15/1) to give the title compound **5c** (201 mg, 83%) as a colourless liquid; R*f* = 0.6 (hexane/ethyl acetate = 15/1). ^1^H NMR (400 MHz, CDCl_3_): δ 7.35–7.19 (m, 9H), 1.31(s, 9H). ^13^C NMR (100 MHz, CDCl_3_): δ 150.4, 136.1, 131.0, 130.4, 130.1, 129.2, 126.4, 126.1, 34.6, 31.4.

### 4-isopropylphenyl)(phenyl)sulfide (5d)^15^

To a 10 mL glass tube, In_2_O_3_ nanoparticles (1.1 mg, 0.004 mmol), TBATB (144 mg, 0.3 mmol), Na_2_CO_3_ (42 mg, 0.4 mmol), thiophenol (22 mg, 0.2 mmol) and 4-isopropylphenylboronic acid (35.6 mg, 0.2 mmol) were dissolved in DMSO (2 mL) under N_2_ atmosphere. The tube was sealed and the mixture was stirred at 110 °C. The reaction mixture was filtered and the solvent evaporated in vacuo to give the crude product, which was purified by column chromatography (hexane/ethyl acetate = 25/1) to give the title compound **5d** (198 mg, 87%) as a colourless liquid; R*f* = 0.6 (hexane/ethyl acetate = 25/1). ^1^H NMR (400 MHz, CDCl_3_): δ 7.44–7.22 (m, 9H), 3.03–2.92 (m, 1H), 1.27 (d, *J* = 7.2 Hz, 6H). ^13^C NMR (100 MHz, CDCl_3_): δ 148.7, 136.7, 131.4, 131.0, 130.6, 129.4, 128.4, 127.3, 127.2, 126.3, 33.6, 23.4.

### (2,4,6-trimethyl-phenyl)-phenyl sulfide (5e)^25^

To a 10 mL glass tube, In_2_O_3_ nanoparticles (1.1 mg, 0.004 mmol), TBATB (144 mg, 0.3 mmol), Na_2_CO_3_ (42 mg, 0.4 mmol), thiophenol (22 mg, 0.2 mmol) and 2,4,6-trimethyl-phenylboronic acid (32.8 mg, 0.2 mmol) were dissolved in DMSO (2 mL) under N_2_ atmosphere. The tube was sealed and the mixture was stirred at 110 °C. The reaction mixture was filtered and the solvent evaporated in vacuo to give the crude product, which was purified by column chromatography (hexane/ethyl acetate = 20/1) to give the title compound **5e** (185 mg, 81%) as a colourless liquid; [Found: C, 78.86; H, 7.17; C_15_H_16_S requires C, 78.90; H, 7.06%]; R*f* = 0.4 (hexane/ethyl acetate = 20/1). ^1^H NMR (400 MHz, CDCl_3_): δ 7.38–7.31 (m, 2H), 7.28–7.18 (m, 3H), 6.96–6.88 (m, 2H) 2.47 (s, 6H), 2.39 (s, 3H). ^13^C NMR (100 MHz, CDCl_3_): δ 143.4, 139.3, 138.4, 129.4, 128.0, 127.7, 125.8, 124.4, 21.6, 21.1.

### 3,5-Dichlorophenyl phenyl sulfide (5f)

To a 10 mL glass tube, In_2_O_3_ nanoparticles (1.1 mg, 0.004 mmol), TBATB (144 mg, 0.3 mmol), Na_2_CO_3_ (42 mg, 0.4 mmol), thiophenol (22 mg, 0.2 mmol) and 3,5-dichlorophenylboronic acid (38 mg, 0.2 mmol) were dissolved in DMSO (2 mL) under N_2_ atmosphere. The tube was sealed and the mixture was stirred at 110 °C. The reaction mixture was filtered and the solvent evaporated in vacuo to give the crude product, which was purified by column chromatography (hexane/ethyl acetate = 15/1) to give the title compound **5f** (140 mg, 55%) as a colourless liquid; R*f* = 0.6 (hexane/ethyl acetate = 15/1). ^1^H NMR (400 MHz, CDCl_3_): δ 7.64–7.54 (m, 5H), 7.37–7.26 (m, 3H). ^13^C NMR (100 MHz, CDCl_3_): δ 140.9, 133.2, 132.7, 131.5, 129.6, 129.3, 128.5, 122.2.

### 3-Nitrophenyl phenyl sulfide (5g)

To a 10 mL glass tube, In_2_O_3_ nanoparticles (1.1 mg, 0.004 mmol), TBATB (144 mg, 0.3 mmol), Na_2_CO_3_ (42 mg, 0.4 mmol), thiophenol (22 mg, 0.2 mmol) and 3-nitrophenylboronic acid (33.4 mg, 0.2 mmol) were dissolved in DMSO (2 mL) under N_2_ atmosphere. The tube was sealed and the mixture was stirred at 110 °C. The reaction mixture was filtered and the solvent evaporated in vacuo to give the crude product, which was purified by column chromatography (hexane/ethyl acetate = 15/1) to give the title compound **5g** (120 mg, 52%) as a pale yellow liquid; R*f* = 0.6 (hexane/ethyl acetate = 15/1). ^1^H NMR (400 MHz, CDCl_3_): δ 7.97–7.78 (m, 4H), 7.44–7.36 (m, 5H). ^13^C NMR (100 MHz, CDCl_3_): δ 134.7, 133.9, 132.3, 129.2, 129.0, 128.9, 128.6, 128.2, 123.4, 120.2.

### 2-Naphthalyl phenyl sulfide (5 h)

To a 10 mL glass tube, In_2_O_3_ nanoparticles (1.1 mg, 0.004 mmol), TBATB (144 mg, 0.3 mmol), Na_2_CO_3_ (42 mg, 0.4 mmol), thiophenol (22 mg, 0.2 mmol) and 2-napthylboronic acid (34.4 mg, 0.2 mmol) were dissolved in DMSO (2 mL) under N_2_ atmosphere. The tube was sealed and the mixture was stirred at 110 °C. The reaction mixture was filtered and the solvent evaporated in vacuo to give the crude product, which was purified by column chromatography (hexane/ethyl acetate = 15/1) to give the title compound **5 h** (184 mg, 78%) as a white solid; mp 50–51 °C; R*f* = 0.5 (hexane/ethyl acetate = 15/1). ^1^H NMR (400 MHz, CDCl_3_): δ 7.86–7.76 (m, 4H), 7.50–7.42 (m, 8H). ^13^C NMR (100 MHz, CDCl_3_): δ 135.7, 133.6, 133.2, 132.7, 130.8, 129.6, 129.4, 128.8, 128.4, 127.4, 127.1, 127.0, 126.9, 126.2.

### 2-(Phenylthio)thiophene (5i)^24^

To a 10 mL glass tube, In_2_O_3_ nanoparticles (1.1 mg, 0.004 mmol), TBATB (144 mg, 0.3 mmol), Na_2_CO_3_ (42 mg, 0.4 mmol), thiophenol (22 mg, 0.2 mmol) and 2-thiopheneboronic acid (25.6 mg, 0.2 mmol) were dissolved in DMSO (2 mL) under N_2_ atmosphere. The tube was sealed and the mixture was stirred at 110 °C. The reaction mixture was filtered and the solvent evaporated in vacuo to give the crude product, which was purified by column chromatography (hexane/ethyl acetate = 15/1) to give the title compound **5i** (163 mg, 85%) as a colourless liquid; [Found: C, 62.59; H, 4.11; C_10_H_8_S_2_ requires C, 62.46; H, 4.19%]; R*f* = 0.6 (hexane/ethyl acetate = 15/1). ^1^H NMR (400 MHz, CDCl_3_): δ 7.51(dd, *J* = 4.4 Hz, 1 H), 7.34–7.22 (m, 6H), 7.16–7.11 (m, 1H). ^13^C NMR (100 MHz, CDCl_3_): δ 138.7, 136.0, 131.5, 131.2, 128.9, 128.1, 127.4, 126.1.

### 3-(Phenylthio)thiophene (5j)^24^

To a 10 mL glass tube, In_2_O_3_ nanoparticles (1.1 mg, 0.004 mmol), TBATB (144 mg, 0.3 mmol), Na_2_CO_3_ (42 mg, 0.4 mmol), thiophenol (22 mg, 0.2 mmol) and 3-thiopheneboronic acid (25.6 mg, 0.2 mmol) were dissolved in DMSO (2 mL) under N_2_ atmosphere. The tube was sealed and the mixture was stirred at 110 °C. The reaction mixture was filtered and the solvent evaporated in vacuo to give the crude product, which was purified by column chromatography (hexane/ethyl acetate = 15/1) to give the title compound **5j** (161 mg, 80%) as a colourless liquid; [Found: C, 62.56; H, 4.09; C_10_H_8_S_2_ requires C, 62.46; H, 4.19%]; R*f* = 0.6 (hexane/ethyl acetate = 15/1). ^1^H NMR (400 MHz, CDCl_3_): δ 7.37–7.34 (m, 2H), 7.26–7.13 (m, 8H), 7.07 (dd, *J* = 8.2 Hz, 1H) ^13^C NMR (100 MHz, CDCl_3_): δ 137.8, 131.6, 129.2, 128.9, 128.6, 128.1, 126.7, 126.0.

## Additional Information

**How to cite this article**: Gogoi, P. *et al.* Role of TBATB in nano indium oxide catalyzed C-S bond formation. *Sci. Rep.* **5**, 13873; doi: 10.1038/srep13873 (2015).

## Supplementary Material

Supplementary Information

## Figures and Tables

**Figure 1:**
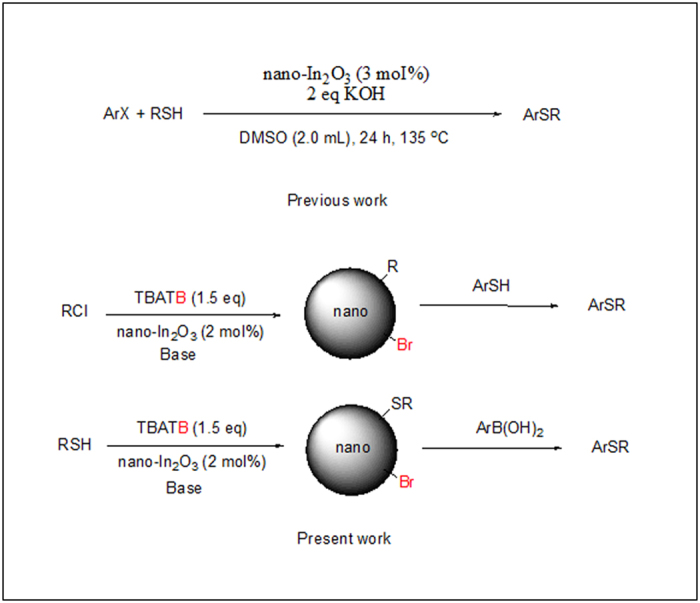
Synthesis of aryl sulfide.

**Table 1:** Optimization of reaction condition for thiophenol to sulfide (Bold examples).

**Entry**	**Catalyst (mol %)**	**Reagent (2eq)(Temp** **°C)**	**Solvent (Base 2eq)**	**Time (h)**	**Yield (%)**^d^
1	Nano-In_2_O_3_ (2)	TBATB (110)	DMSO(Na_2_CO_3_)	2	99^a^
		TBATB (110)	DMSO(Na_2_CO_3_)	2	99^b^
		TBATB (110)	DMSO(Na_2_CO_3_)	4	45^c^
		------- (110)	DMSO(Na_2_CO_3_)	18	75
		TBATB (rt)	DMSO(Na_2_CO_3_)	10	46
2	Nano In_2_O_3_ (2)	Br_2_ (110)	DMSO(NaOH)	2	40
3	Nano-In_2_O_3_ (5)	TBATB (110)	DMSO(Na_2_CO_3_)	2	95
4	Nano-In_2_O_3_ (2)	TBATB (130)	CH_2_Cl_2_ (KOH)	4	65
5	Nano-Co_3_O_4_(5)	TBATB (110)	CH_3_COOH(Na_2_CO_3_)	4	40
6	Nano-Co_3_O_4_ (10)	TBATB (110)	CH_3_CN(Na_2_CO_3_)	2	64

^a^Reaction was carried out at mmol scale using 0.2 mmol (22 mg) of thiophenol, (2 moI%) catalyst, bromine source (1.5 equiv) and base (2.0 equiv).

^b^Reaction was also carried out at gram scale using 1.10 g of thiophenol, (2 moI%) catalyst and TBATB (1.5 equiv), Na_2_CO_3_ (2.0 equiv).

^c^Without catalyst.

^d^Isolated yield.
