# Supplementary material for: Role of TBATB in nano indium oxide catalyzed C-S bond formation
Source: Sci Rep. 2015 Sep 29;5:13873. doi: 10.1038/srep13873 (PMC4586445; doi:10.1038/srep13873)
Supplement: Supplementary Information [file srep13873-s1.pdf]

SUPPORTING INFORMATION

Role of TBATB in nano Indium oxide catalyzed C-S bond formation

Prasanta Gogoi*, Sukanya Hazarika and Pranjit Barman

Department of Chemistry, National Institute of Technology, Silchar 788010, Assam, India

General Information

All solvents and chemicals were purchased commercially and used without further purification. Melting points were recorded on an electrothermal digital melting point apparatus and were uncorrected. Column chromatography was generally performed on silica gel (230-400 mesh) and reactions were monitored by thin layer chromatography (TLC) using UV light (254 nm) to visualize the course of reactions. ^{1}H and ^{13}C Nuclear Magnetic Resonance spectra of pure compounds were acquired at 400 and 100 MHz respectively. All NMR samples were recorded in deuterated chloroform. Chemical shifts (ppm) were recorded with tetramethylsilane (TMS) as the internal reference standard. Elemental analyses were performed on a Flash 2000 Thermo Scientific instrument at NIT Silchar. The TEM and SEM characterization were carried out at model no. CM-12 Philips TEM (IIT Kharagpur) and FEI Nova nanoSEM-450 (NIT Rourkela) respectively.

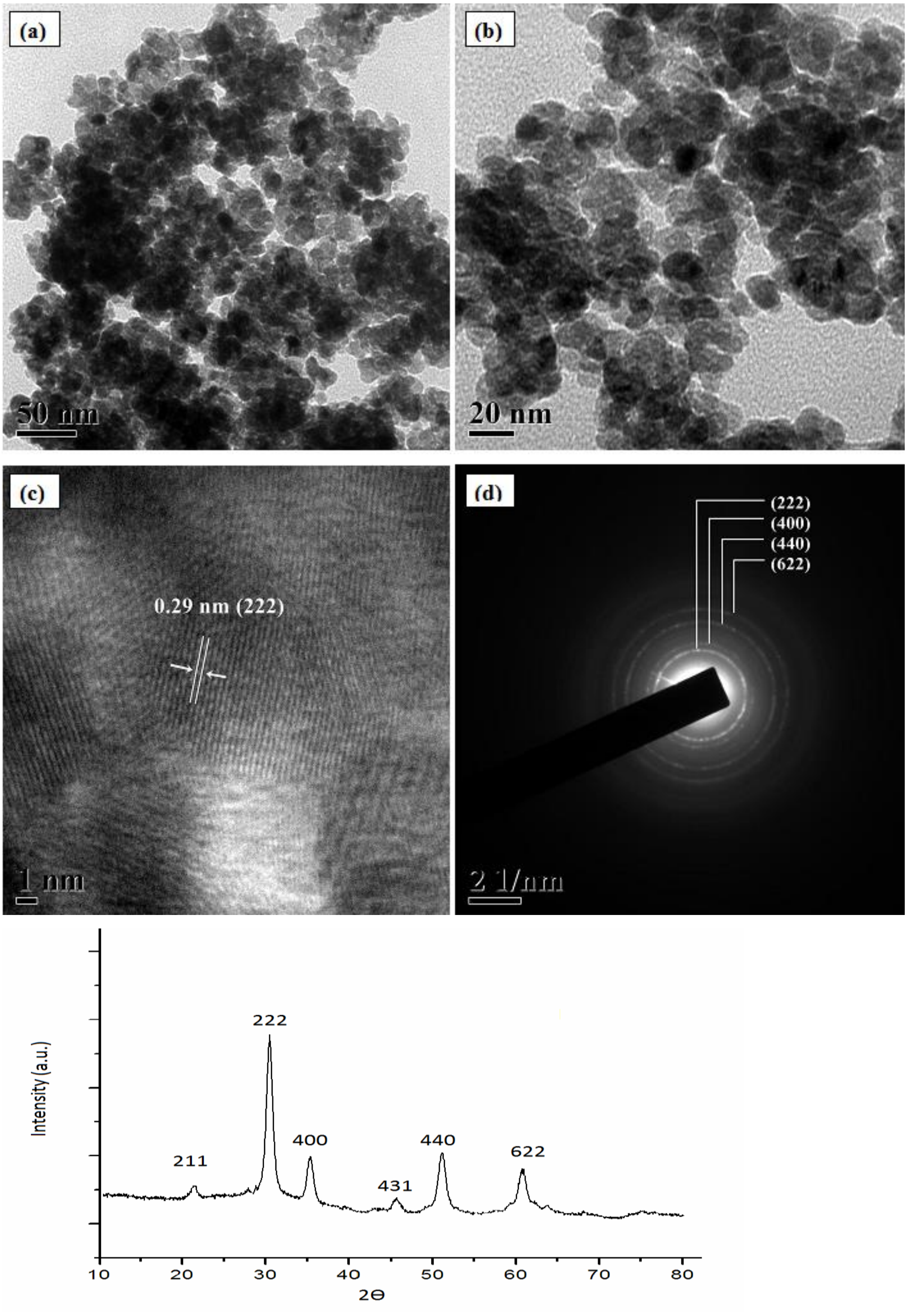

Figure 2. TEM-images of In_2O_3 nanoparticles at **(a-b)** lower and **(c)** higher magnification **(d)** SAED pattern **(e)** powder XRD

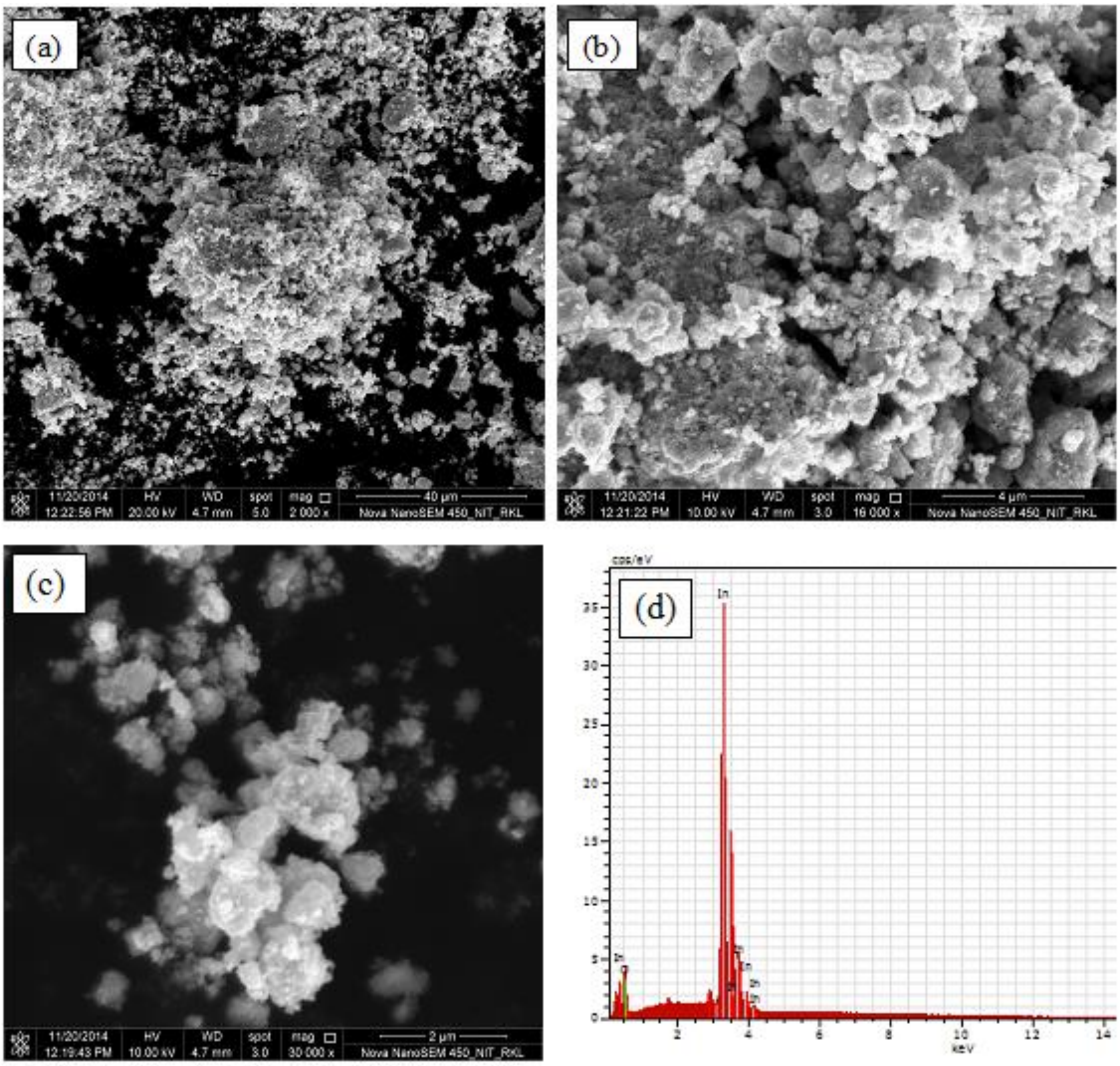

Figure 3. SEM-images of In_2O_3 nanoparticles at **(a-b)** lower and **(c)** higher magnification **(d)** Energy dispersive X-ray spectroscopy (EDS)

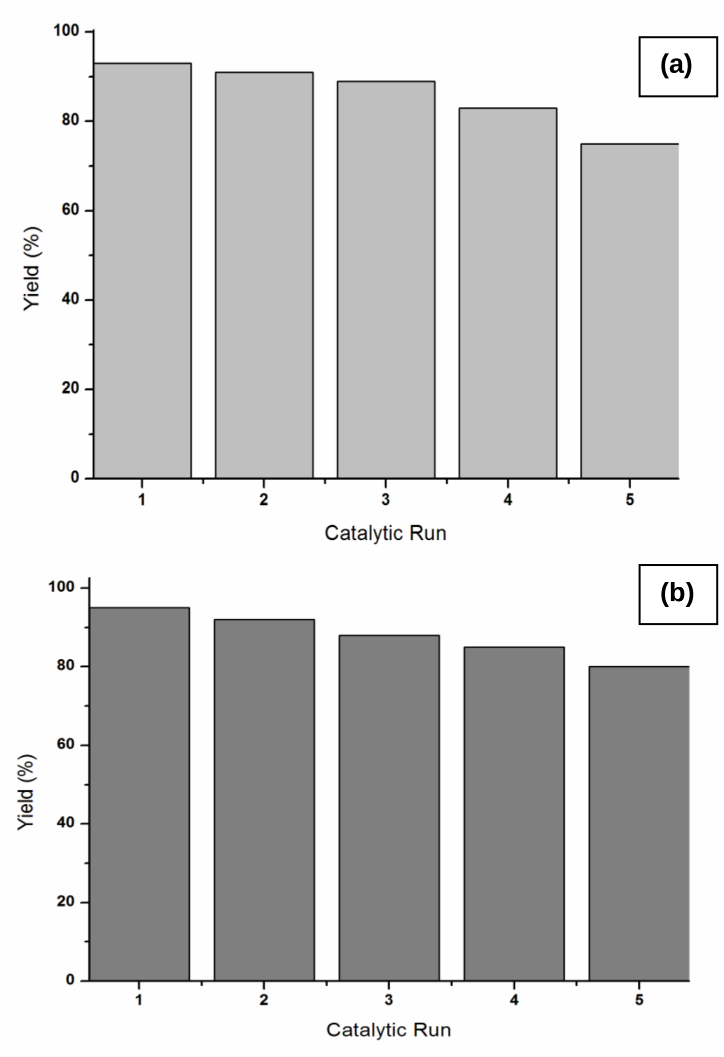

Figure 8. Recovery of In_2O_3 nps: (a) Reactions were carried out with allyl chloride (0.2 mmol) phenylthiol (0.2 mmol), nano-In_2O_3 (2 mol%), TBATB (1.5 equiv), Na_2CO_3 (2.0 equiv) in DMSO (2 mL) for 2h. **(b)** Reactions were carried out with phenylboronic acid (0.2 mmol), phenylthiol (0.2 mmol), nano-In_2O_3 (2 mol%), TBATB (1.5 equiv), Na_2CO_3 (2.0 equiv) in DMSO (2 mL) for 2h.

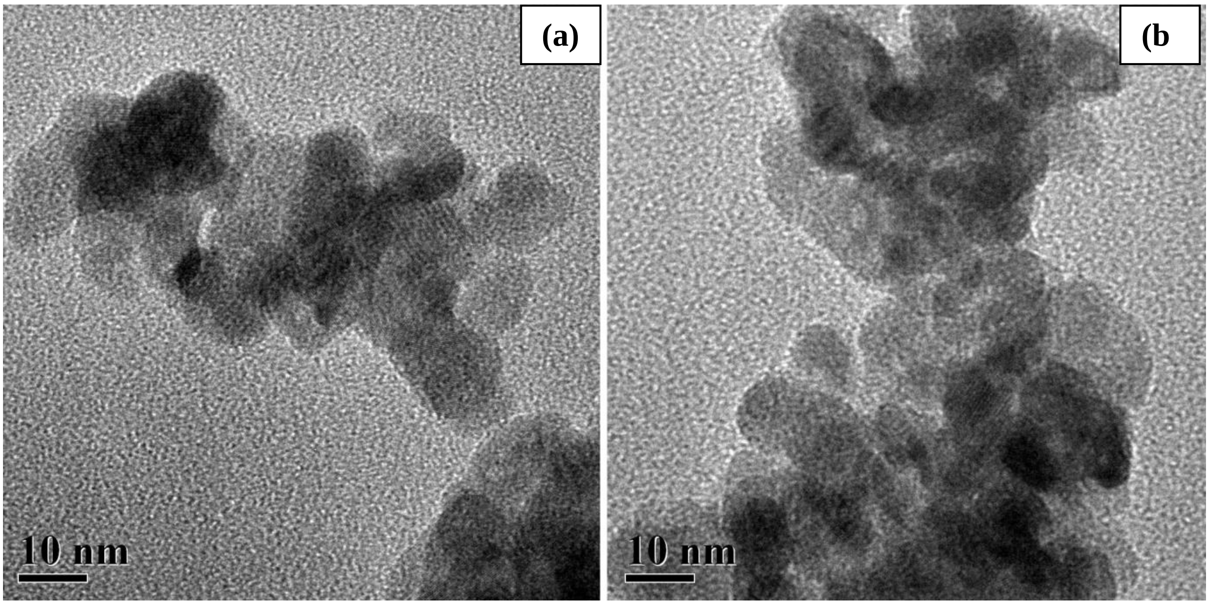

Figure 9. TEM images of recovered In_2O_3 nps after 5th cycle: (a) Reactions were carried out with allyl chloride (0.2 mmol) phenylthiol (0.2 mmol), nano-In_2O_3 (2 mol%), TBATB (1.5 equiv), Na_2CO_3 (2.0 equiv) in DMSO (2 mL) for 2h. **(b)** Reactions were carried out with phenyIboronic acid (0.2 mmol), phenyIthiol (0.2 mmol), nano-In_2O_3 (2 mol%), TBATB (1.5 equiv), Na_2CO_3 (2.0 equiv) in DMSO (2 mL) for 2h.

Allyl(phenyl)sulfane (3a)[19]

To a 10 mL glass tube, In_2O_3 nanoparticles (1.1 mg, 0.004 mmol), TBATB (144 mg, 0.3 mmol), Na_2CO_3 (42 mg, 0.4 mmol), thiophenol (22 mg, 0.2 mmol) and allyl chloride (15.2 mg, 0.2 mmol) were dissolved in DMSO (2 mL) under N_2 atmosphere. The tube was sealed and the mixture was stirred at the corresponding temperature for 2h. The reaction mixture was filtered and the solvent evaporated in vacuo to give the crude product, which was purified by column chromatography (hexane/ethyl acetate = 25/1) to give the title compound **3a** (149 mg, 99%) as a light yellow liquid; [Found: C, 71.98; H, 6.49; $C_9H_{10}S$. requires C, 71.95; H, 6.71%]; R*f* = 0.4 (hexane/ethyl acetate = 20/1). ^1H NMR (400 MHz, $CDCl_3$): δ 7.39-7.28 (m, 5H), 5.94 (m, 1H), 5.28 (dd, *J* = 1.3 Hz, *J* = 16.4 Hz, 1H), 5.13 (dd, *J* = 1.2 Hz, *J* = 10.1 Hz, 1H),3.68 (d, *J* = 6.8 Hz, 2H) ^{13}C NMR (100 MHz, $CDCl_3$): δ 134.9, 133.9, 130.7, 129.8, 126.9, 118.9, 37.4.

(2-methylallyl)(phenyl)sulfane (3c)[23]

To a 10 mL glass tube, In_2O_3 nanoparticles (1.1 mg, 0.004 mmol), TBATB (144 mg, 0.3 mmol), Na_2CO_3 (42 mg, 0.4 mmol), thiophenol (22 mg, 0.2 mmol) and 2-chloro-2-methyl-propene (18.2 mg, 0.2 mmol) were dissolved in DMSO (2 mL) under N_2 atmosphere. The tube was sealed and the mixture was stirred at the corresponding temperature for 2h. The reaction mixture was filtered and the solvent evaporated in vacuo to give the crude product, which was purified by column chromatography (hexane/ethyl acetate = 10/1) to give the title compound **3c** (114.8 mg, 70%) as a colourless liquid; [Found: C, 72.98; H, 7.39; $C_{10}H_{12}S$ requires C, 73.12; H, 7.36%]; R*f* = 0.4 (hexane/ethyl acetate = 10/1). ^1H NMR (400 MHz, $CDCl_3$): δ 7.44-7.33 (m, 5H), 5.16 (d, *J* = 1.2 Hz, 1H), 4.89 (d, *J* = 1.1 Hz, 1H), 3.65 (s, 2H), 1.87 (s, 3H). ^{13}C NMR (100 MHz, $CDCl_3$): δ 142.4, 135.3, 131.2, 129.1, 128.2, 112.5, 42.9, 21.7.

(2-bromoallyl)(phenyl)sulfane (3d)[24]

To a 10 mL glass tube, In_2O_3 nanoparticles (1.1 mg, 0.004 mmol), TBATB (144 mg, 0.3 mmol), Na_2CO_3 (42 mg, 0.4 mmol), thiophenol (22 mg, 0.2 mmol) and 2-bromo-allyl bromide (39.6 mg, 0.2 mmol) were dissolved in DMSO (2 mL) under N_2 atmosphere. The tube was sealed and the mixture was stirred at the corresponding temperature for 2h. The reaction mixture was filtered and the solvent evaporated in vacuo to give the crude product, which was purified by column chromatography (hexane/ethyl acetate = 15/1) to give the title compound **3d** (201 mg, 88%) as a colourless liquid; [Found: C, 47.16; H, 3.93; S, 14.04; C_9H_9BrS requires C, 47.18; H, 3.96; S, 13.99 %]; R*f* = 0.4 (hexane/ethyl acetate = 15/1). ^1H NMR (400 MHz, $CDCl_3$): δ 7.53-7.30 (m, 5H), 5.89 (d, *J*=1.8 Hz, 1H), 5.50 (d, *J* = 1.7 Hz, 1H), 4.01 (m, 2H). ^{13}C NMR (100 MHz, $CDCl_3$): δ 135.2,131.1, 129.4, 129.2, 127.5, 119.6, 45.2.

(2-chloroallyl)(phenyl)sulfane (3e)

To a 10 mL glass tube, In_2O_3 nanoparticles (1.1 mg, 0.004 mmol), TBATB (144 mg, 0.3 mmol), Na_2CO_3 (42 mg, 0.4 mmol), thiophenol (22 mg, 0.2 mmol) and 2-chloro-allyl chloride (22 mg, 0.2 mmol) were dissolved in DMSO (2 mL) under N_2 atmosphere. The tube was sealed and the mixture was stirred at the corresponding temperature for 2h. The reaction mixture was filtered and the solvent evaporated in vacuo to give the crude product, which was purified by column chromatography (hexane/ethyl acetate = 20/1) to give the title compound **3e** (153 mg, 83%) as a colourless liquid; [Found: C, 58.58; H, 4.80; C_9H_9ClS

requires C, 58.53; H, 4.91%]; R*f* = 0.4 (hexane/ethyl acetate = 20/1). ^{1}H NMR (400 MHz, $CDCl_3$): δ 7.94-7.86 (m, 5H), 5.77-5.64 (m, 2H), 3.88 (d, *J* = 6.3 Hz, 2H). ^{13}C NMR (100 MHz, $CDCl_3$): δ 140.8, 137.7, 133.9, 128.4, 126.2, 119.7, 44.1.

Allyl(*p*-tolyl)sulfane (3g)[22]

To a 10 mL glass tube, In_2O_3 nanoparticles (1.1 mg, 0.004 mmol), TBATB (144 mg, 0.3 mmol), Na_2CO_3 (42 mg, 0.4 mmol), 4-methylthiophenol (24.8 mg, 0.2 mmol) and allyl chloride (15.2 mg, 0.2 mmol) were dissolved in DMSO (2 mL) under N_2 atmosphere. The tube was sealed and the mixture was stirred at the corresponding temperature for 2h. The reaction mixture was filtered and the solvent evaporated in vacuo to give the crude product, which was purified by column chromatography (hexane/ethyl acetate = 15/1) to give the title compound **3g** (144 mg, 88%) as a colourless liquid; [Found: C, 73.13; H, 7.31. $C_{10}H_{12}S$. requires C, 73.12; H, 7.36 %]; R*f* = 0.5 (hexane/ethyl acetate = 10/1). ^{1}H NMR (400 MHz, $CDCl_3$): δ 7.38 (d, *J* = 8.1 Hz, 2H), 7.03 (d, *J* = 7.9 Hz, 2H), 5. 74-5.55 (m, 1H), 5.16-5.06 (m, 2H), 3.58 (d, *J* = 7.6 Hz, 2H), 2.35 (s, 3H).^{13}C NMR (100 MHz, $CDCl_3$): δ 138.7, 134.2, 132.8,131.6, 129.5, 117.9, 37.2, 20.1

allyl(3-chlorophenyl)sulfane (3h)

To a 10 mL glass tube, In_2O_3 nanoparticles (1.1 mg, 0.004 mmol), TBATB (144 mg, 0.3 mmol), Na_2CO_3 (42 mg, 0.4 mmol), 3-chlorothiophenol (28.8 mg, 0.2 mmol) and allyl chloride (15.2 mg, 0.2 mmol) were dissolved in DMSO (2 mL) under N_2 atmosphere. The tube was sealed and the mixture was stirred at the corresponding temperature for 2h. The reaction mixture was filtered and the solvent evaporated in vacuo to give the crude product, which was purified by column chromatography (hexane/ethyl acetate = 15/1) to give the title compound **3h** (105 mg, 65%) as a yellow liquid; [Found: C, 58.58; H, 4.94; C_9H_9ClS requires C, 58.53; H, 4.91 %]; R*f* = 0.5 (hexane/ethyl acetate = 15/1). ^{1}H NMR (400 MHz, $CDCl_3$): δ 7.38-7.29 (m, 4H), 5.86-5.75 (m, 1H), 5.27 (dd, *J* = 1.1 Hz, *J* = 16.1 Hz, 1H), 5.17 (dd, *J* = 1.3 Hz, *J* = 10.2 Hz, 1H), 3.59 (d, *J* = 6.1 Hz, 2H). ^{13}C NMR (100 MHz, $CDCl_3$): δ 137.7, 134.2, 132.5, 128.9, 127.9, 125.6, 124.3, 117.7, 35.9.

Allyl(4-nitrophenyl)sulfane (3i)[21]

To a 10 mL glass tube, In_2O_3 nanoparticles (1.1 mg, 0.004 mmol), TBATB (144 mg, 0.3 mmol), Na_2CO_3 (42 mg, 0.4 mmol), 4-nitrothiophenol (31 mg, 0.2 mmol) and allyl chloride (15.2 mg, 0.2 mmol) were dissolved in DMSO (2 mL) under N_2 atmosphere. The tube was sealed and the mixture was stirred at the corresponding temperature for 2h. The reaction mixture was filtered and the solvent evaporated in vacuo to give the crude product, which was purified by column chromatography (hexane/ethyl acetate = 15/1) to give the title compound **3i** (105 mg, 54%) as a yellow solid, mp 39-41 ^{o}C; [Found: C, 55.31; H, 4.66; N, 6.99; $C_9H_9NO_2S$ requires C, 55.37; H, 4.65; N, 7.17 %]; R*f* = 0.6 (hexane/ethyl acetate = 15/1). ^{1}H NMR (400 MHz, $CDCl_3$): δ 8.45 (d, J = 8.1 Hz, 2H), 7.38 (d, J = 8.6 Hz, 2H), 5.89-5.83 (m, 1H), 5.36 (dd, J = 1.1 Hz, J = 16.3 Hz, 1H), 5.27 (dd, J = 1.4 Hz, J = 10.1 Hz, 1H), 3.76 (d, J = 6.3 Hz, 2H). ^{13}C NMR (100 MHz, $CDCl_3$): δ 147.8, 146.7, 133.9, 127.8, 124.8, 119.4, 35.2.

(2-bromoallyl)(4-nitrophenyl)sulfane (3j)

To a 10 mL glass tube, In_2O_3 nanoparticles (1.1 mg, 0.004 mmol), TBATB (144 mg, 0.3 mmol), Na_2CO_3 (42 mg, 0.4 mmol), 4-nitrothiophenol (31 mg, 0.2 mmol) and 2-bromo-allyl bromide (39.6 mg, 0.2 mmol) were dissolved in DMSO (2 mL) under N_2 atmosphere.

The tube was sealed and the mixture was stirred at the corresponding temperature for 2h. The reaction mixture was filtered and the solvent evaporated in vacuo to give the crude product, which was purified by column chromatography (hexane/ethyl acetate = 10/1) to give the title compound **3j** (167 mg, 61%) as a colourless liquid; [Found: C, 39.49; H, 2.99; N, 5.01; $C_9H_8BrNO_2S$ requires C, 39.43; H, 2.94; N, 5.11%]; R*f* = 0.4 (hexane/ethyl acetate = 10/1). ^{1}H NMR (400 MHz, $CDCl_3$): δ 8.20 (d, *J* = 9.1 Hz, 2H), 7.36 (d, *J* = 8.2 Hz, 2H), 5.77 (d, *J* = 2.9 Hz, 1H), 5.47 (d, *J* = 2.6 Hz, 1H), 4.01 (s, 2H). ^{13}C NMR (100 MHz, $CDCl_3$): δ 143.1, 139.2, 125.7, 124.9, 121.1, 44.8.

Diphenyl sulfide (5a)[15]

To a 10 mL glass tube, In_2O_3 nanoparticles (1.1 mg, 0.004 mmol), TBATB (144 mg, 0.3 mmol), Na_2CO_3 (42 mg, 0.4 mmol), thiophenol (22 mg, 0.2 mmol) and phenylboronic acid (24.4 mg, 0.2 mmol) were dissolved in DMSO (2 mL) under N_2 atmosphere. The tube was sealed and the mixture was stirred at 110 ^{o}C. The reaction mixture was filtered and the solvent evaporated in vacuo to give the crude product, which was purified by column chromatography (hexane/ethyl acetate = 15/1) to give the title compound **5a** (177 mg, 95%) as a colourless liquid; [Found: 77.55; H, 5.47; $C_{12}H_{10}S$ requires C, 77.37; H, 5.41%]; R*f* = 0.4 (hexane/ethyl acetate = 15/1). ^{1}H NMR (400 MHz, $CDCl_3$): δ 7.40 – 7.27 (m, 10H). ^{13}C NMR (100 MHz, $CDCl_3$): δ 135.5, 131.2, 129.2, 127.7 .

2, 6-Dimethylphenyl phenyl sulfide (5b)[13]

To a 10 mL glass tube, In_2O_3 nanoparticles (1.1 mg, 0.004 mmol), TBATB (144 mg, 0.3 mmol), Na_2CO_3 (42 mg, 0.4 mmol), thiophenol (22 mg, 0.2 mmol) and 2,6-dimethyl-phenylboronic acid (30 mg, 0.2 mmol) were dissolved in DMSO (2 mL) under N_2 atmosphere. The tube was sealed and the mixture was stirred at 110 ^{o}C. The reaction mixture was filtered and the solvent evaporated in vacuo to give the crude product, which was purified by column chromatography (hexane/ethyl acetate = 20/1) to give the title compound **5b** (180 mg, 84%) as a colourless liquid; [Found: C, 78.65; H, 6.54; C14H14S requires C, 78.45; H, 6.58%]; R*f* = 0.4 (hexane/ethyl acetate = 20/1). ^{1}H NMR (400 MHz, $CDCl_3$): δ

7.28-7.22 (m, 5H), 7.10– 6.96 (m, 3H), 2.46 (s, 6H). ^{13}C NMR (100 MHz, $CDCl_3$): δ 144.1, 138.2, 130.7, 129.4, 128.4, 128.0, 125.3, 124.0, 21.4.

4-*tert*-Butylphenyl phenyl sulfide (5c)

To a 10 mL glass tube, In_2O_3 nanoparticles (1.1 mg, 0.004 mmol), TBATB (144 mg, 0.3 mmol), Na_2CO_3 (42 mg, 0.4 mmol), thiophenol (22 mg, 0.2 mmol) and 4-*tert*-butylphenylboronic acid (35.6 mg, 0.2 mmol were dissolved in DMSO (2 mL) under N_2 atmosphere. The tube was sealed and the mixture was stirred at 110 ^oC. The reaction mixture was filtered and the solvent evaporated in vacuo to give the crude product, which was purified by column chromatography (hexane/ethyl acetate = 15/1) to give the title compound **5c** (201 mg, 83%) as a colourless liquid; R*f* = 0.6 (hexane/ethyl acetate = 15/1). ^{1}H NMR (400 MHz, $CDCl_3$): δ 7.35-7.19 (m, 9H), 1.31(s, 9H). ^{13}C NMR (100 MHz, $CDCl_3$): δ 150.4, 136.1, 131.0, 130.4, 130.1, 129.2, 126.4, 126.1, 34.6, 31.4.

4-isopropylphenyl)(phenyl)sulfide (5d)[11]

To a 10 mL glass tube, In_2O_3 nanoparticles (1.1 mg, 0.004 mmol), TBATB (144 mg, 0.3 mmol), Na_2CO_3 (42 mg, 0.4 mmol), thiophenol (22 mg, 0.2 mmol) and 4-isopropylphenylboronic acid (35.6 mg, 0.2 mmol) were dissolved in DMSO (2 mL) under N_2 atmosphere. The tube was sealed and the mixture was stirred at 110 ^oC. The reaction mixture was filtered and the solvent evaporated in vacuo to give the crude product, which was purified by column chromatography (hexane/ethyl acetate = 25/1) to give the title compound **5d** (198 mg, 87%) as a colourless liquid; R*f* = 0.6 (hexane/ethyl acetate = 25/1). ^{1}H NMR (400 MHz, $CDCl_3$): δ 7.44-7.22 (m, 9H), 3.03–2.92 (m, 1H), 1.27 (d, *J* = 7.2 Hz, 6H). ^{13}C NMR (100 MHz, $CDCl_3$): δ 148.7, 136.7, 131.4, 131.0, 130.6, 129.4, 128.4, 127.3, 127.2, 126.3, 33.6, 23.4.

.

(2,4,6-trimethyl-phenyl)-phenyl sulfide (5e)[18]

To a 10 mL glass tube, In_2O_3 nanoparticles (1.1 mg, 0.004 mmol), TBATB (144 mg, 0.3 mmol), Na_2CO_3 (42 mg, 0.4 mmol), thiophenol (22 mg, 0.2 mmol) and 2,4,6-trimethyl-phenylboronic acid (32.8 mg, 0.2 mmol) were dissolved in DMSO (2 mL) under N_2

atmosphere. The tube was sealed and the mixture was stirred at 110 °C. The reaction mixture was filtered and the solvent evaporated in vacuo to give the crude product, which was purified by column chromatography (hexane/ethyl acetate = 20/1) to give the title compound **5e** (185 mg, 81%) as a colourless liquid; [Found: C, 78.86; H, 7.17; $C_{15}H_{16}S$ requires C, 78.90; H, 7.06%]; R*f* = 0.4 (hexane/ethyl acetate = 20/1). ^1H NMR (400 MHz, $CDCl_3$): δ 7.38-7.31 (m, 2H), 7.28-7.18 (m, 3H), 6.96-6.88 (m, 2H) 2.47 (s, 6H), 2.39 (s, 3H). ^{13}C NMR (100 MHz, $CDCl_3$): δ 143.4, 139.3, 138.4, 129.4, 128.0, 127.7, 125.8, 124.4, 21.6, 21.1.

3,5-Dichlorophenyl phenyl sulfide (5f)

To a 10 mL glass tube, In_2O_3 nanoparticles (1.1 mg, 0.004 mmol), TBATB (144 mg, 0.3 mmol), Na_2CO_3 (42 mg, 0.4 mmol), thiophenol (22 mg, 0.2 mmol) and 3,5-dichlorophenylboronic acid (38 mg, 0.2 mmol) were dissolved in DMSO (2 mL) under N_2 atmosphere. The tube was sealed and the mixture was stirred at 110 °C. The reaction mixture was filtered and the solvent evaporated in vacuo to give the crude product, which was purified by column chromatography (hexane/ethyl acetate = 15/1) to give the title compound **5f** (140 mg, 55%) as a colourless liquid; R*f* = 0.6 (hexane/ethyl acetate = 15/1). ^1H NMR (400 MHz, $CDCl_3$): δ 7.64-7.54 (m, 5H), 7.37-7.26 (m, 3H). ^{13}C NMR (100 MHz, $CDCl_3$): δ 140.9, 133.2, 132.7, 131.5, 129.6, 129.3, 128.5, 122.2.

.

3-Nitrophenyl phenyl sulfide (5g)

To a 10 mL glass tube, In_2O_3 nanoparticles (1.1 mg, 0.004 mmol), TBATB (144 mg, 0.3 mmol), Na_2CO_3 (42 mg, 0.4 mmol), thiophenol (22 mg, 0.2 mmol) and 3-nitrophenylboronic acid (33.4 mg, 0.2 mmol) were dissolved in DMSO (2 mL) under N_2 atmosphere. The tube was sealed and the mixture was stirred at 110 °C. The reaction mixture was filtered and the solvent evaporated in vacuo to give the crude product, which was purified by column chromatography (hexane/ethyl acetate = 15/1) to give the title compound **5g** (120 mg, 52%) as a pale yellow liquid; R*f* = 0.6 (hexane/ethyl acetate = 15/1). ^1H NMR (400 MHz, $CDCl_3$): δ 7.97-7.78 (m, 4H), 7.44-7.36 (m, 5H). ^{13}C NMR (100 MHz, $CDCl_3$): δ 134.7, 133.9, 132.3, 129.2, 129.0, 128.9, 128.6, 128.2, 123.4, 120.2.

2-Naphthalyl phenyl sulfide (5h)

To a 10 mL glass tube, In_2O_3 nanoparticles (1.1 mg, 0.004 mmol), TBATB (144 mg, 0.3 mmol), Na_2CO_3 (42 mg, 0.4 mmol), thiophenol (22 mg, 0.2 mmol) and 2-

napthylboronic acid (34.4 mg, 0.2 mmol) were dissolved in DMSO (2 mL) under N_2 atmosphere. The tube was sealed and the mixture was stirred at 110 ^{o}C. The reaction mixture was filtered and the solvent evaporated in vacuo to give the crude product, which was purified by column chromatography (hexane/ethyl acetate = 15/1) to give the title compound **5h** (184 mg, 78%) as a white solid**;** mp 50−51 ^{o}C; R*f* = 0.5 (hexane/ethyl acetate = 15/1). ^{1}H NMR (400 MHz, $CDCl_3$): δ 7.86−7.76 (m, 4H), 7.50−7.42 (m, 8H). ^{13}C NMR (100 MHz, $CDCl_3$): δ 135.7, 133.6, 133.2, 132.7, 130.8, 129.6, 129.4, 128.8, 128.4, 127.4, 127.1, 127.0, 126.9, 126.2.

.

2-(Phenylthio)thiophene (5i)[13]

To a 10 mL glass tube, In_2O_3 nanoparticles (1.1 mg, 0.004 mmol), TBATB (144 mg, 0.3 mmol), Na_2CO_3 (42 mg, 0.4 mmol), thiophenol (22 mg, 0.2 mmol) and 2-thiopheneboronic acid (25.6 mg, 0.2 mmol) were dissolved in DMSO (2 mL) under N_2 atmosphere. The tube was sealed and the mixture was stirred at 110 ^{o}C. The reaction mixture was filtered and the solvent evaporated in vacuo to give the crude product, which was purified by column chromatography (hexane/ethyl acetate = 15/1) to give the title compound **5i** (163 mg, 85%) as a colourless liquid; [Found: C, 62.59; H, 4.11; $C_{10}H_8S_2$ requires C, 62.46; H, 4.19%]; R*f* = 0.6 (hexane/ethyl acetate = 15/1). ^{1}H NMR (400 MHz, $CDCl_3$): δ 7.51(dd, *J* = 4.4Hz, 1H), 7.34-7.22 (m, 6H), 7.16-7.11 (m, 1H). ^{13}C NMR (100 MHz, $CDCl_3$): δ 138.7, 136.0, 131.5, 131.2, 128.9, 128.1, 127.4, 126.1.

3-(Phenylthio)thiophene (5j)[13]

To a 10 mL glass tube, In_2O_3 nanoparticles (1.1 mg, 0.004 mmol), TBATB (144 mg, 0.3 mmol), Na_2CO_3 (42 mg, 0.4 mmol), thiophenol (22 mg, 0.2 mmol) and 3-thiopheneboronic acid (25.6 mg, 0.2 mmol) were dissolved in DMSO (2 mL) under N_2 atmosphere. The tube was sealed and the mixture was stirred at 110 ^{o}C. The reaction mixture was filtered and the solvent evaporated in vacuo to give the crude product, which was purified by column chromatography (hexane/ethyl acetate = 15/1) to give the title compound **5j** (161 mg, 80%) as a colourless liquid; [Found: C, 62.56; H, 4.09; $C_{10}H_8S_2$ requires C,

62.46; H, 4.19%]; R*f* = 0.6 (hexane/ethyl acetate = 15/1). ^{1}H NMR (400 MHz, $CDCl_3$): δ 7.37-7.34 (m, 2H), 7.26-7.13 (m, 8H), 7.07(dd, *J* = 8.2Hz, 1H) ^{13}C NMR (100 MHz, $CDCl_3$): δ 137.8, 131.6, 129.2, 128.9, 128.6, 128.1, 126.7, 126.0.

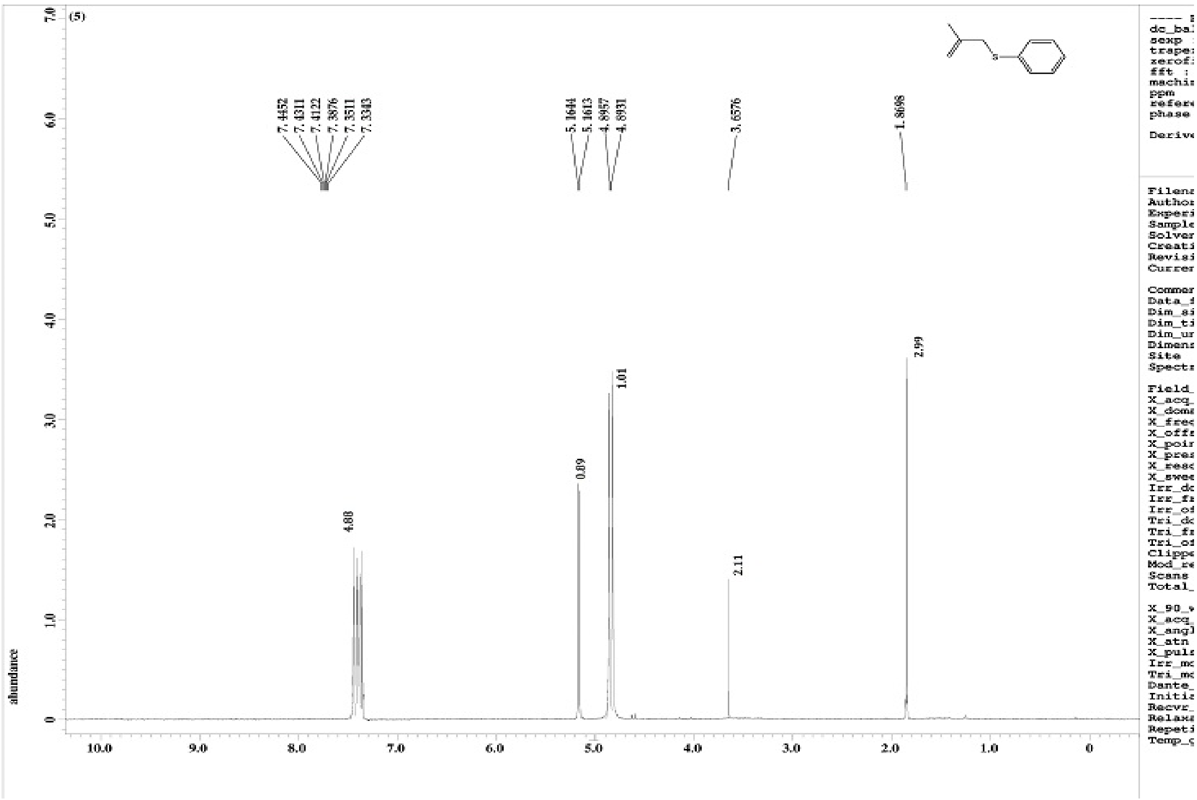

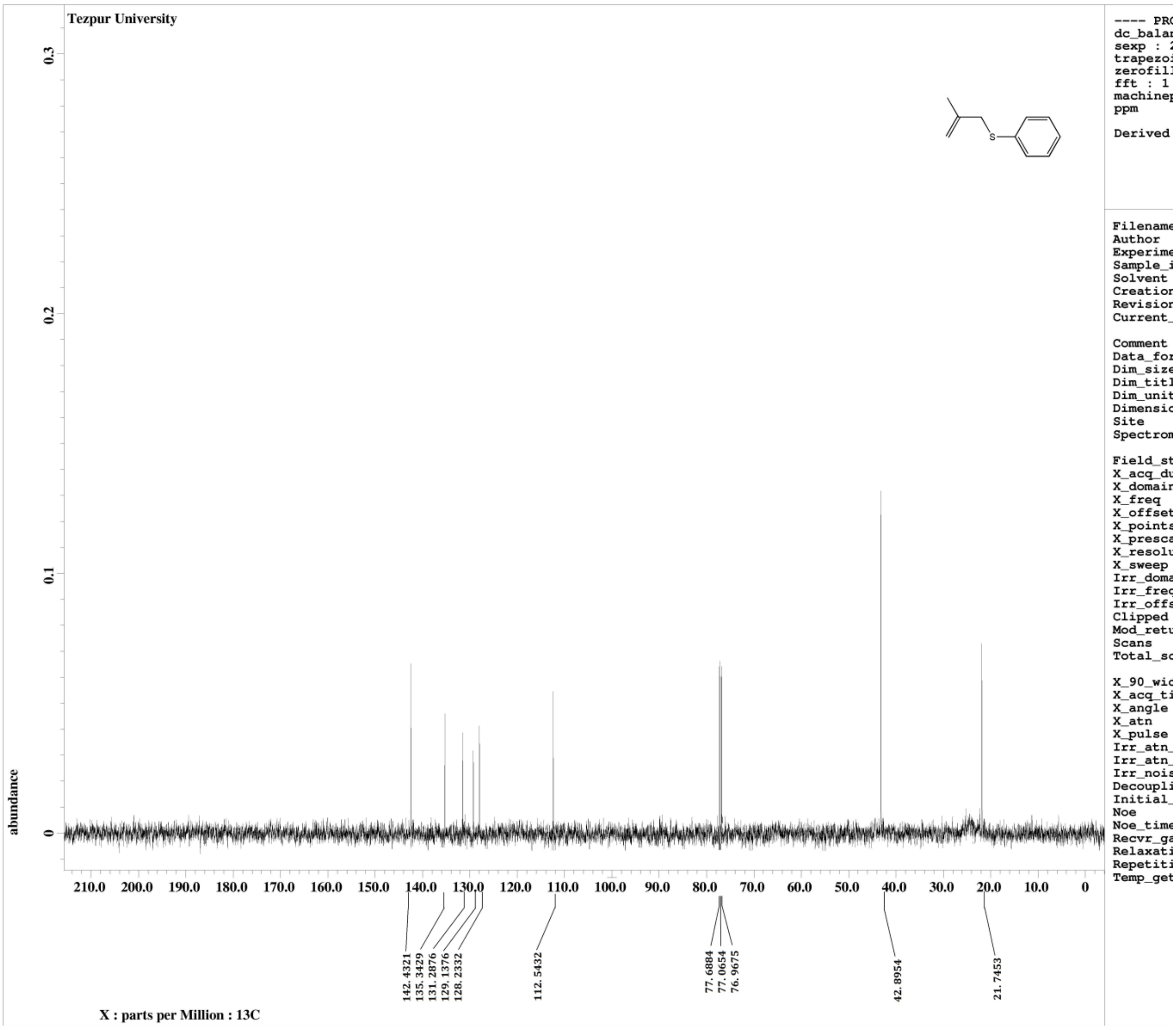
Tezpur University
abundance
X : parts per Million : 13C
142.4321
135.3429
131.2876
129.1376
128.2332
112.5432
77.6884
77.0654
76.9675
42.8954
21.7453

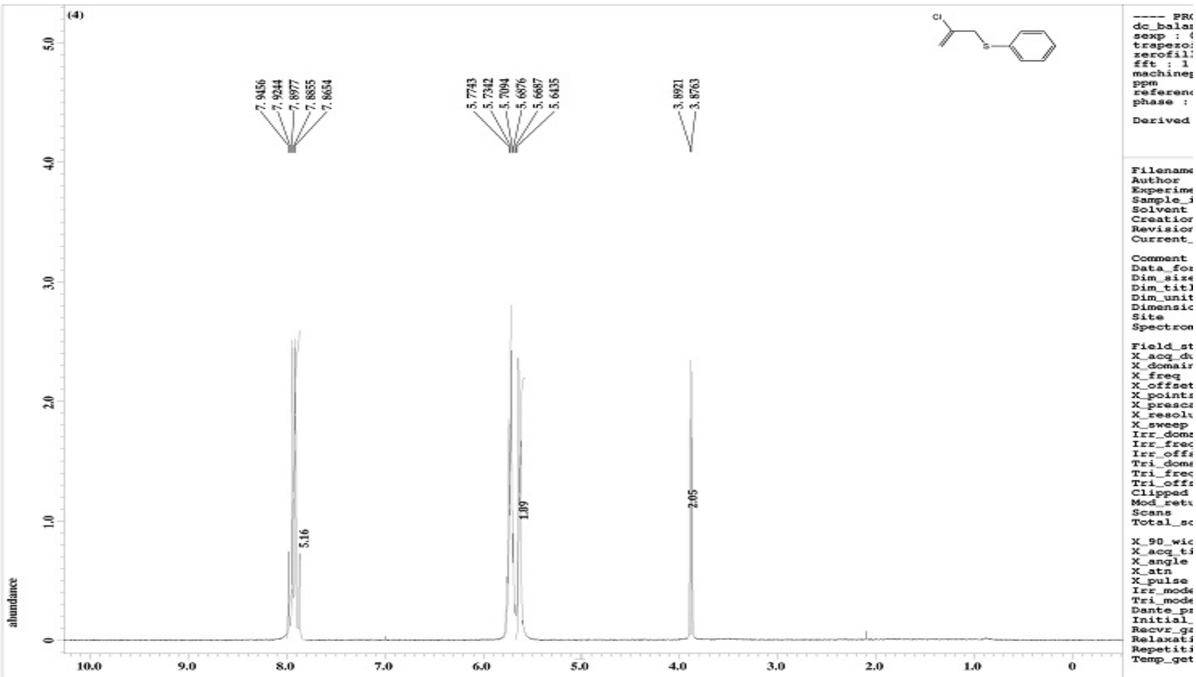
(4)
7.9456
7.9244
7.8977
7.8855
7.8654
5.7743
5.7342
5.7094
5.6876
5.6687
5.6435
3.8921
3.8763
5.16
1.89
2.05
abundance

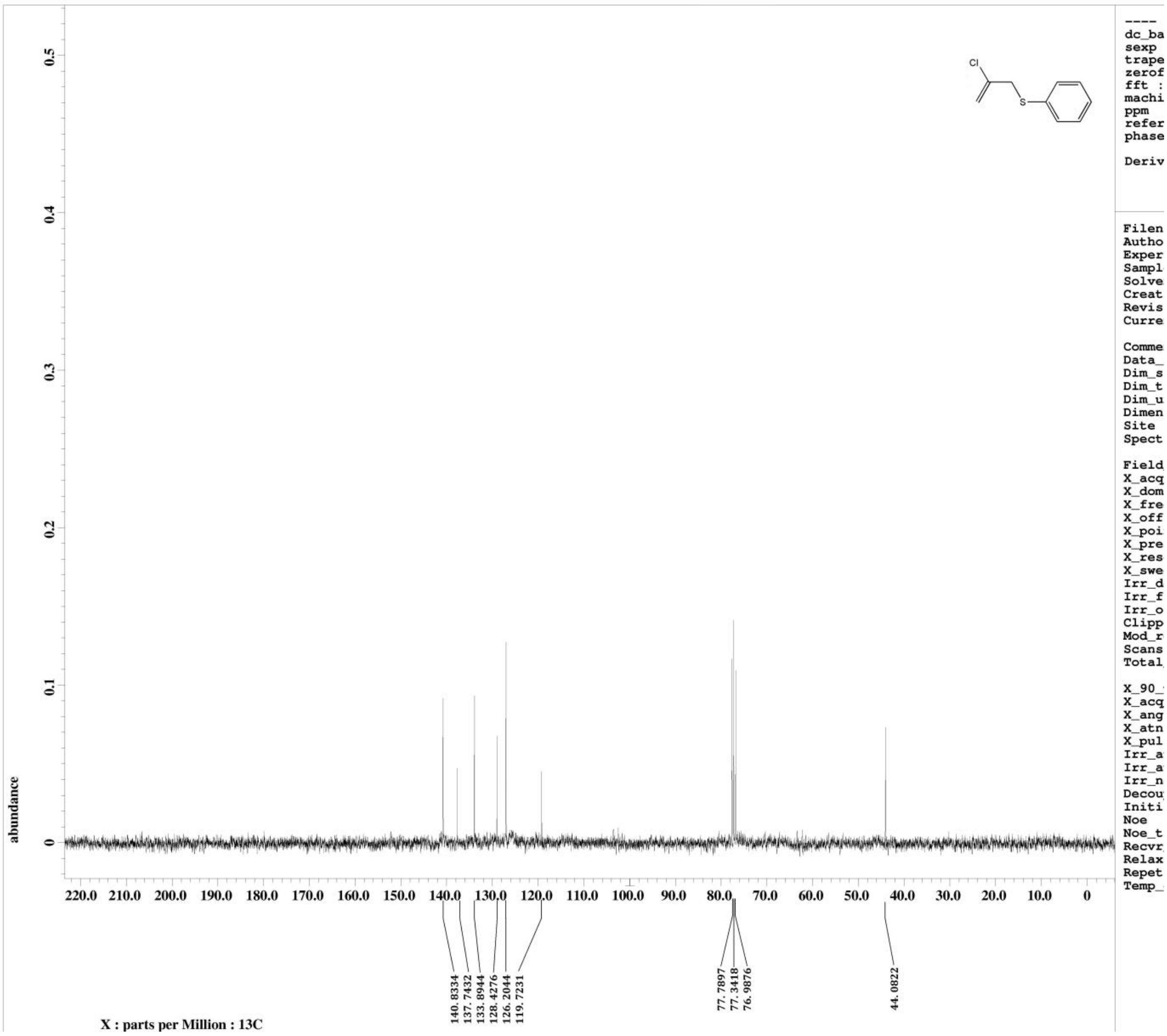
Cl
S
abundance
0.5
0.4
0.3
0.2
0.1
0
220.0 210.0 200.0 190.0 180.0 170.0 160.0 150.0 140.0 130.0 120.0 110.0 100.0 90.0 80.0 70.0 60.0 50.0 40.0 30.0 20.0 10.0 0
140.8334
137.7432
133.8944
128.4276
126.2044
119.7231
77.7897
77.3418
76.9876
44.0822
X : parts per Million : 13C
----
dc_ba
sexp
trape
zerof
fft :
machi
ppm
refer
phase
Deriv
Filen
Autho
Exper
Sampl
Solve
Creat
Revis
Curre
Comme
Data_
Dim_s
Dim_t
Dim_u
Dimen
Site
Spect
Field
X_acq
X_dom
X_fre
X_off
X_poi
X_pre
X_res
X_swe
Irr_d
Irr_f
Irr_o
Clipp
Mod_r
Scans
Total
X_90_
X_acq
X_ang
X_atn
X_pul
Irr_a
Irr_a
Irr_n
Decou
Initi
Noe
Noe_t
Recvr
Relax
Repet
Temp_

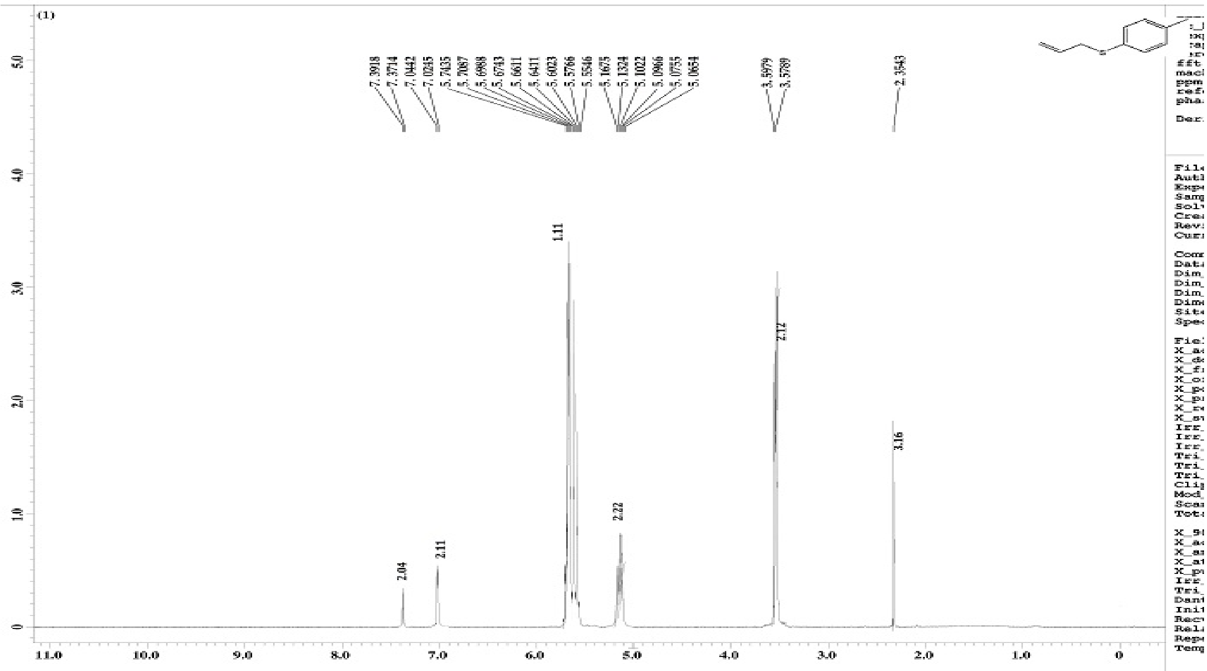
(1)
7.3918
7.3714
7.0442
7.0245
5.7435
5.7087
5.6988
5.6743
5.6611
5.6411
5.6023
5.5766
5.5546
5.1675
5.1324
5.1022
5.0966
5.0755
5.0654
3.5979
3.5789
2.3543
1.11
2.12
3.16
2.22
2.11
2.04

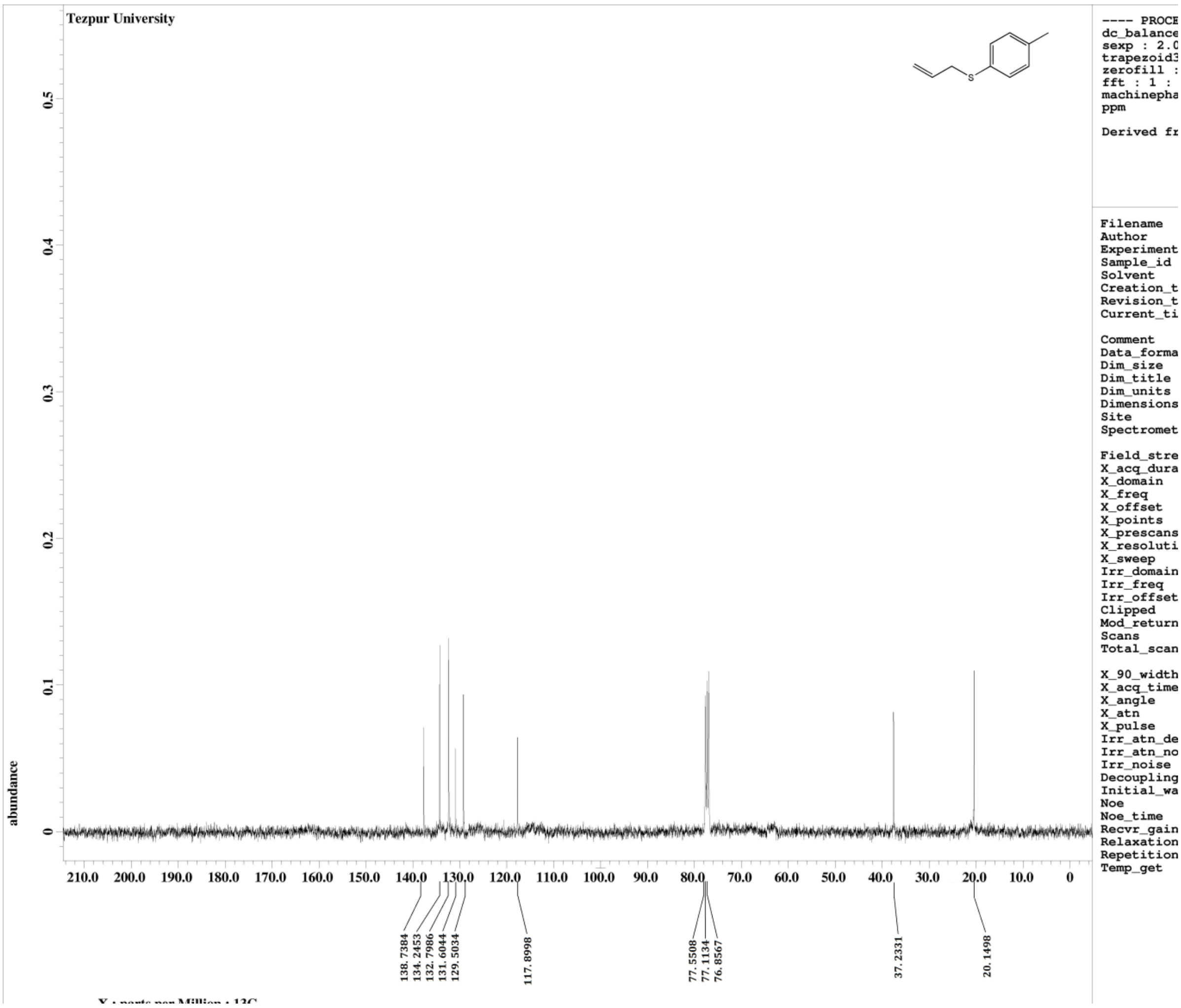
Tezpur University
abundance
138.7384
134.2453
132.7986
131.6044
129.5034
117.8998
77.5508
77.1134
76.8567
37.2331
20.1498

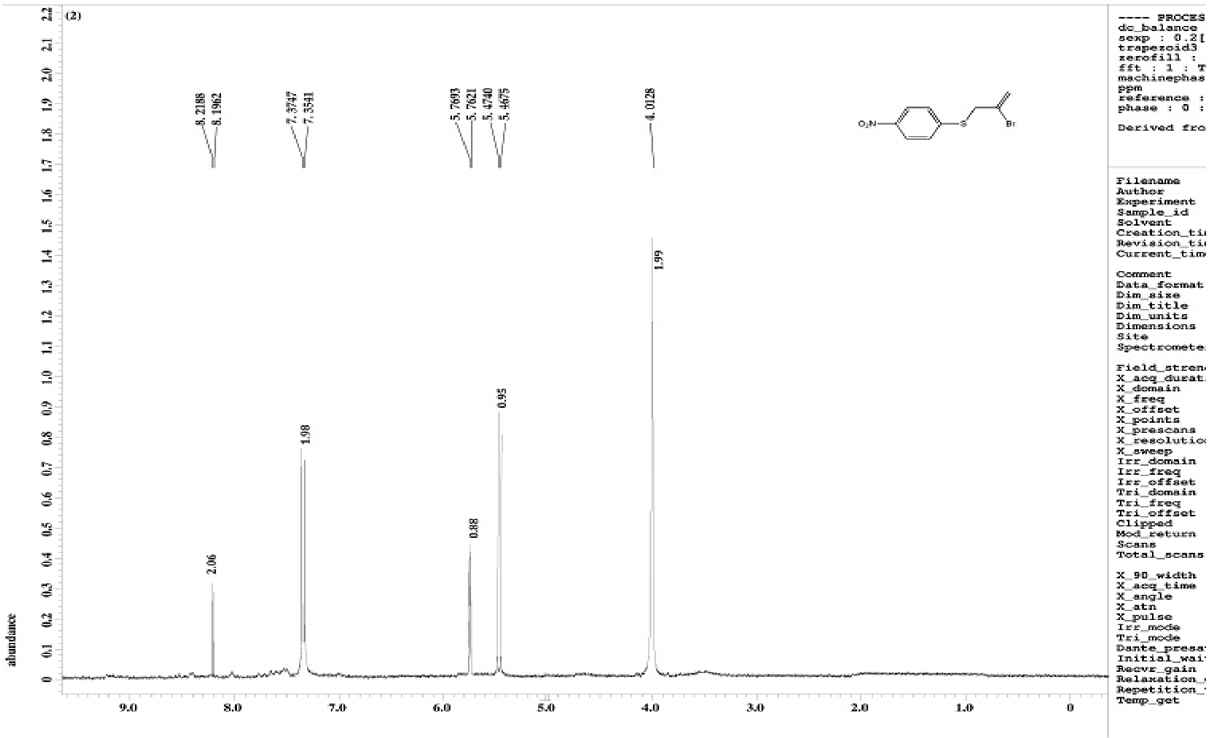
(2)
8.2188
8.1962
7.3747
7.3541
5.7693
5.7621
5.4740
5.4675
4.0128
2.06
1.98
0.88
0.95
1.99
abundance
9.0
8.0
7.0
6.0
5.0
4.0
3.0
2.0
1.0
0
Br
---- PROCES
dc_balance
sexp : 0.2[
trapezoid3
zerofill :
fft : 1 : T
machinephas
ppm
reference :
phase : 0 :
Derived fro
Filename
Author
Experiment
Sample_id
Solvent
Current_tim
Comment
Data_format
Dim_size
Dim_title
Dim_units
Dimensions
Site
X_domain
X_freq
X_offset
X_points
X_prescans
X_sweep
Irr_domain
Irr_freq
Irr_offset
Tri_domain
Tri_freq
Tri_offset
Clipped
Mod_return
Scans
Total_scans
X_90_width
X_acq_time
X_angle
X_atn
X_pulse
Irr_mode
Tri_mode
Recvr_gain
Temp_get

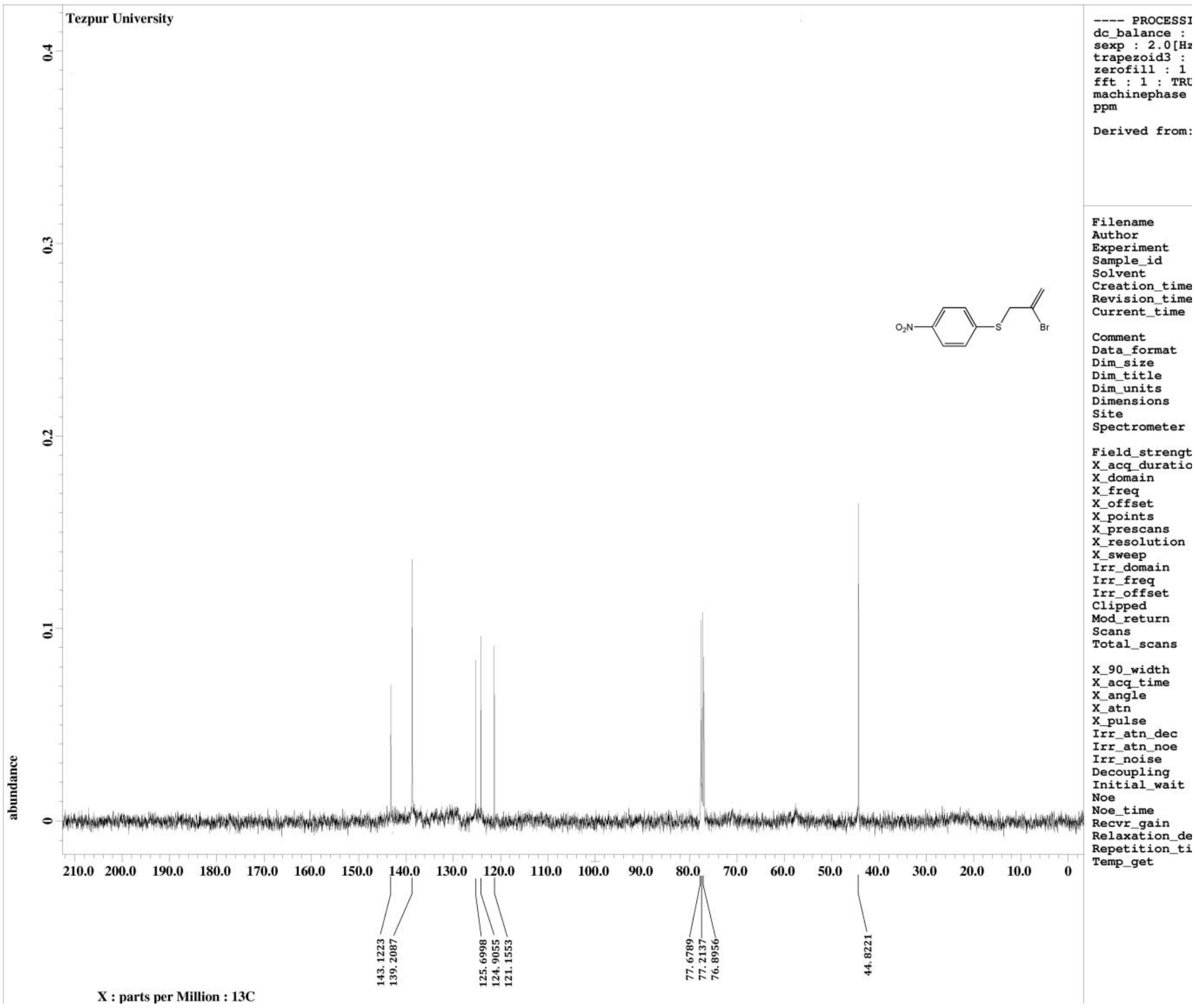
Tezpur University
---- PROCESSI
dc_balance :
sexp : 2.0[Hz
trapezoid3 :
zerofill : 1
fft : 1 : TRU
machinephase
ppm
Derived from:
Filename
Author
Experiment
Sample_id
Solvent
Creation_time
Revision_time
Current_time
Comment
Data_format
Dim_size
Dim_title
Dim_units
Dimensions
Site
Spectrometer
Field_strengt
X_acq_duratio
X_domain
X_freq
X_offset
X_points
X_prescans
X_resolution
X_sweep
Irr_domain
Irr_freq
Irr_offset
Clipped
Mod_return
Scans
Total_scans
X_90_width
X_acq_time
X_angle
X_atn
X_pulse
Irr_atn_dec
Irr_atn_noe
Irr_noise
Decoupling
Initial_wait
Noe
Noe_time
Recvr_gain
Relaxation_de
Repetition_ti
Temp_get
O2N
S
Br
abundance
0.4
0.3
0.2
0.1
0
210.0 200.0 190.0 180.0 170.0 160.0 150.0 140.0 130.0 120.0 110.0 100.0 90.0 80.0 70.0 60.0 50.0 40.0 30.0 20.0 10.0 0
143.1223
139.2087
125.6998
124.9055
121.1553
77.6789
77.2137
76.8956
44.8221
X : parts per Million : 13C

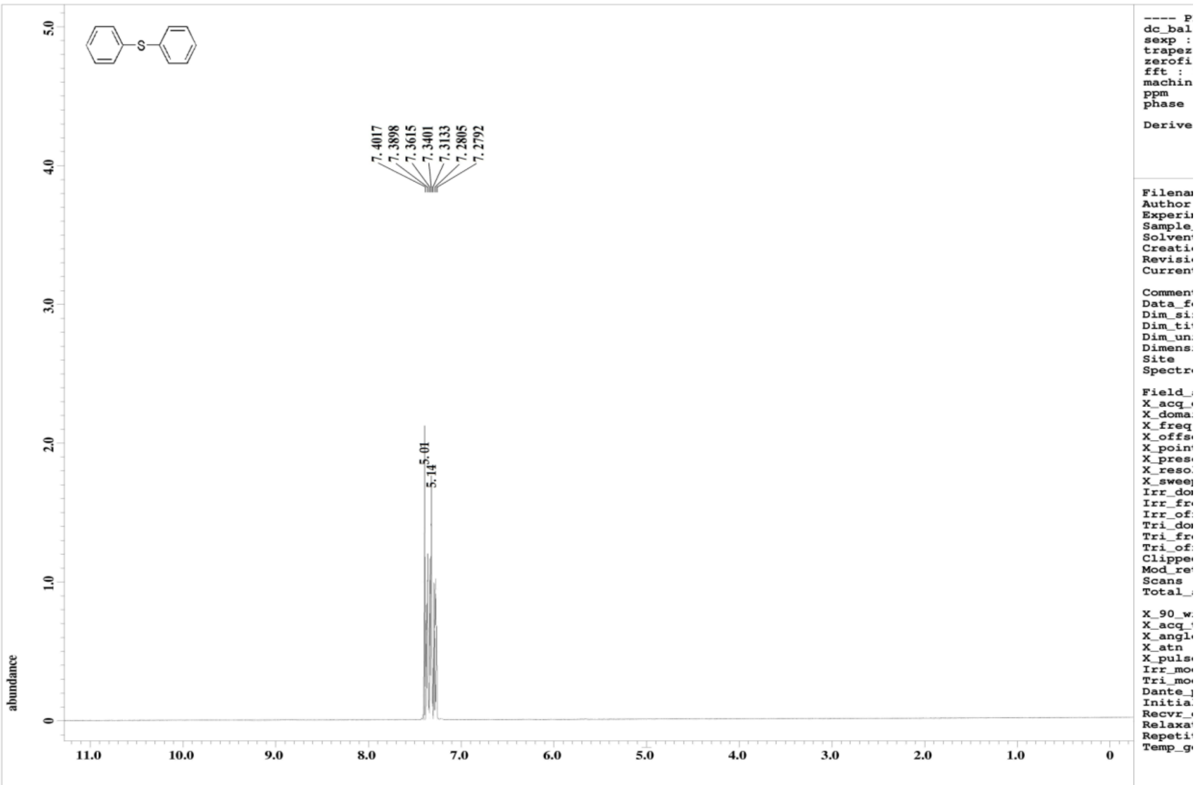

S
7.4017
7.3898
7.3615
7.3401
7.3133
7.2805
7.2792
5.01
5.14
abundance
11.0
10.0
9.0
8.0
7.0
6.0
5.0
4.0
3.0
2.0
1.0
0

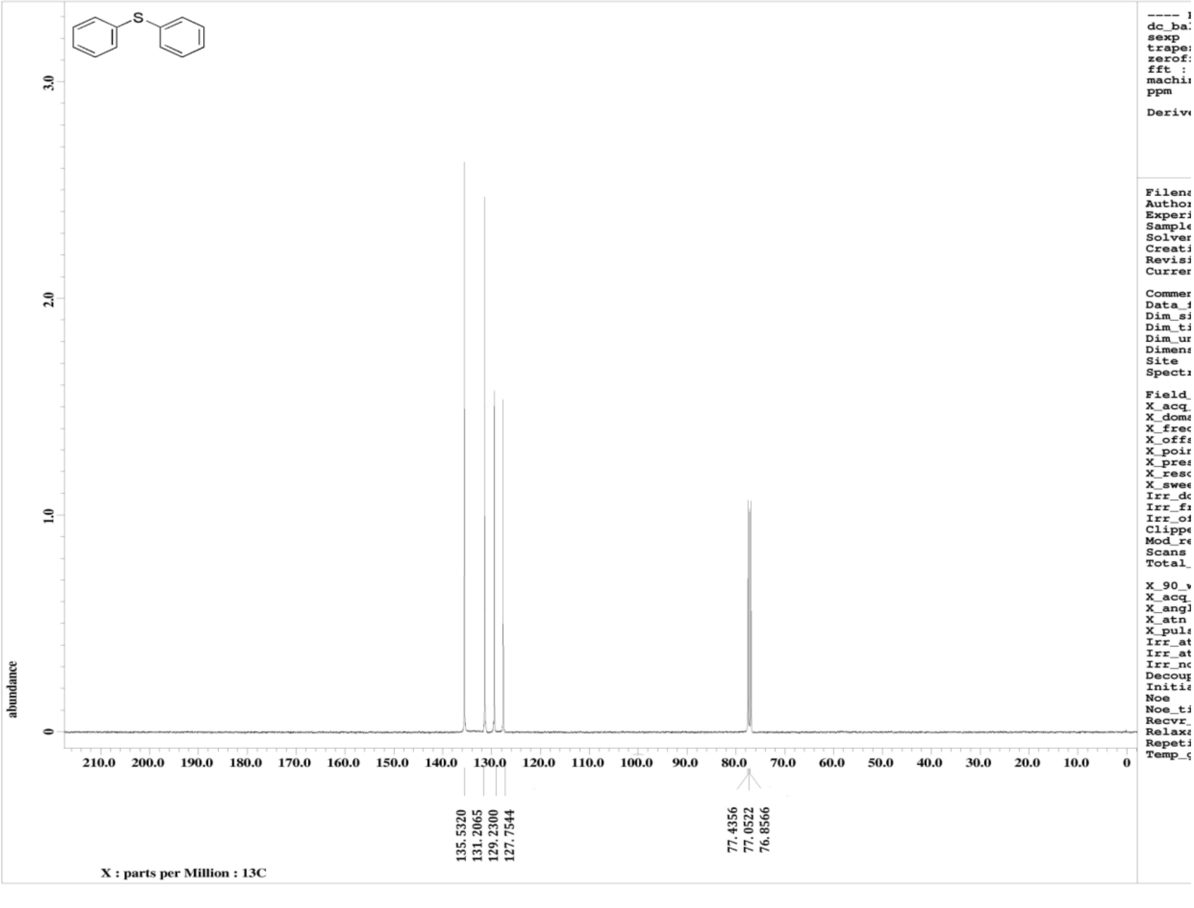

S
abundance
135.5320
131.2065
129.2300
127.7544
77.4356
77.0522
76.8566
X : parts per Million : 13C

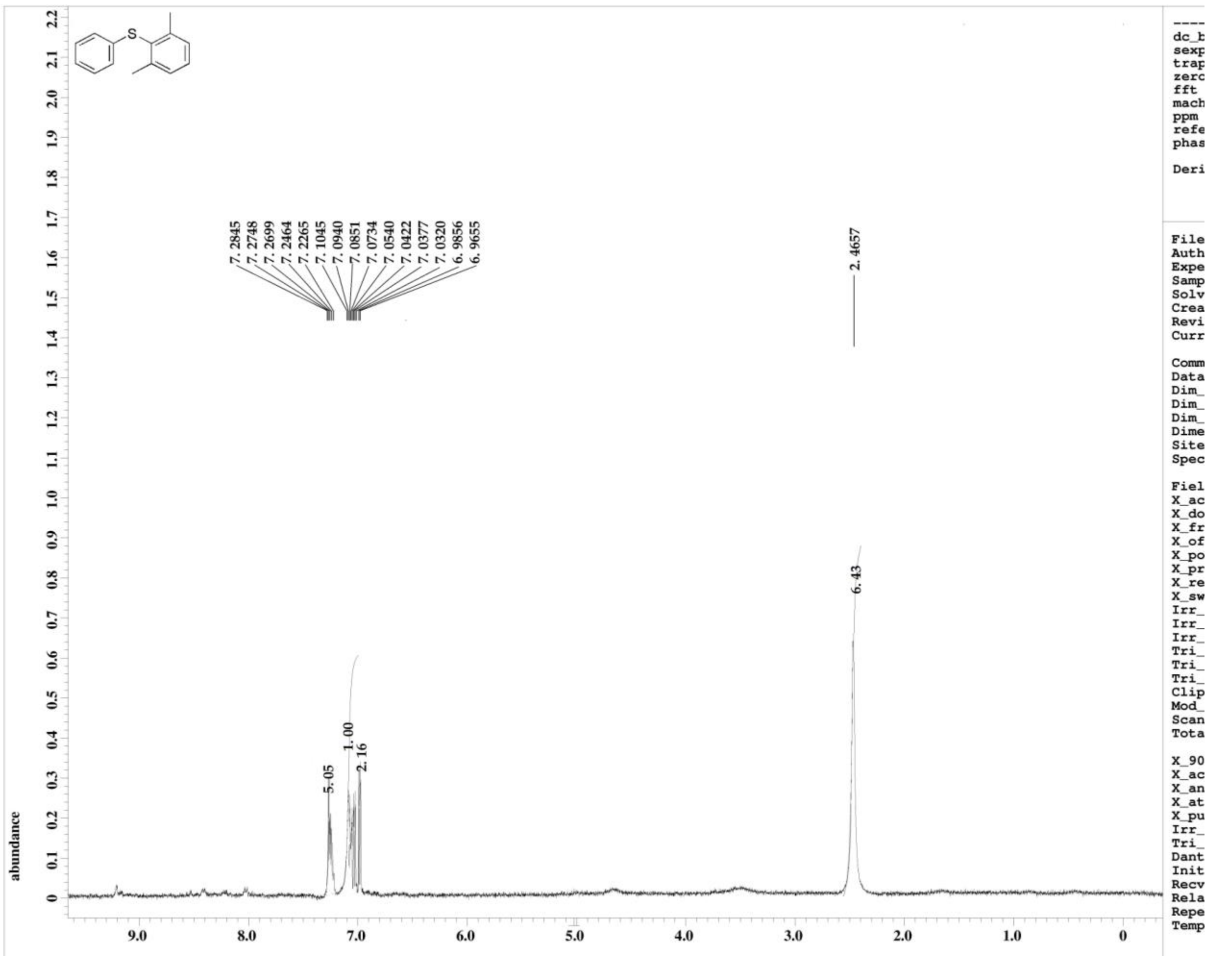
S
7.2845
7.2748
7.2699
7.2464
7.2265
7.1045
7.0940
7.0851
7.0734
7.0540
7.0422
7.0377
7.0320
6.9856
6.9655
2.4657
5.05
1.00
2.16
6.43
abundance

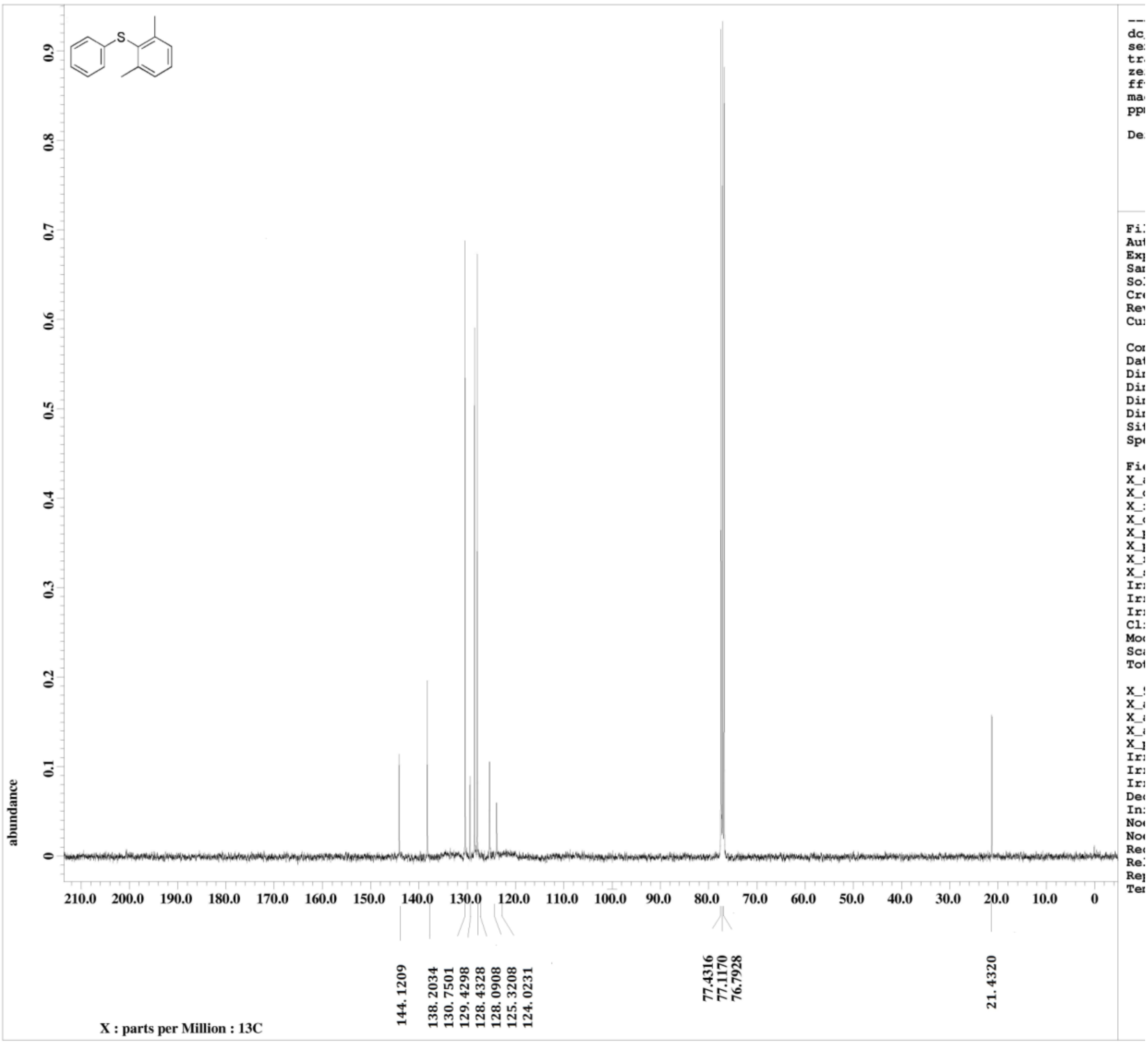
abundance
X : parts per Million : 13C
144.1209
138.2034
130.7501
129.4298
128.4328
128.0908
125.3208
124.0231
77.4316
77.1170
76.7928
21.4320

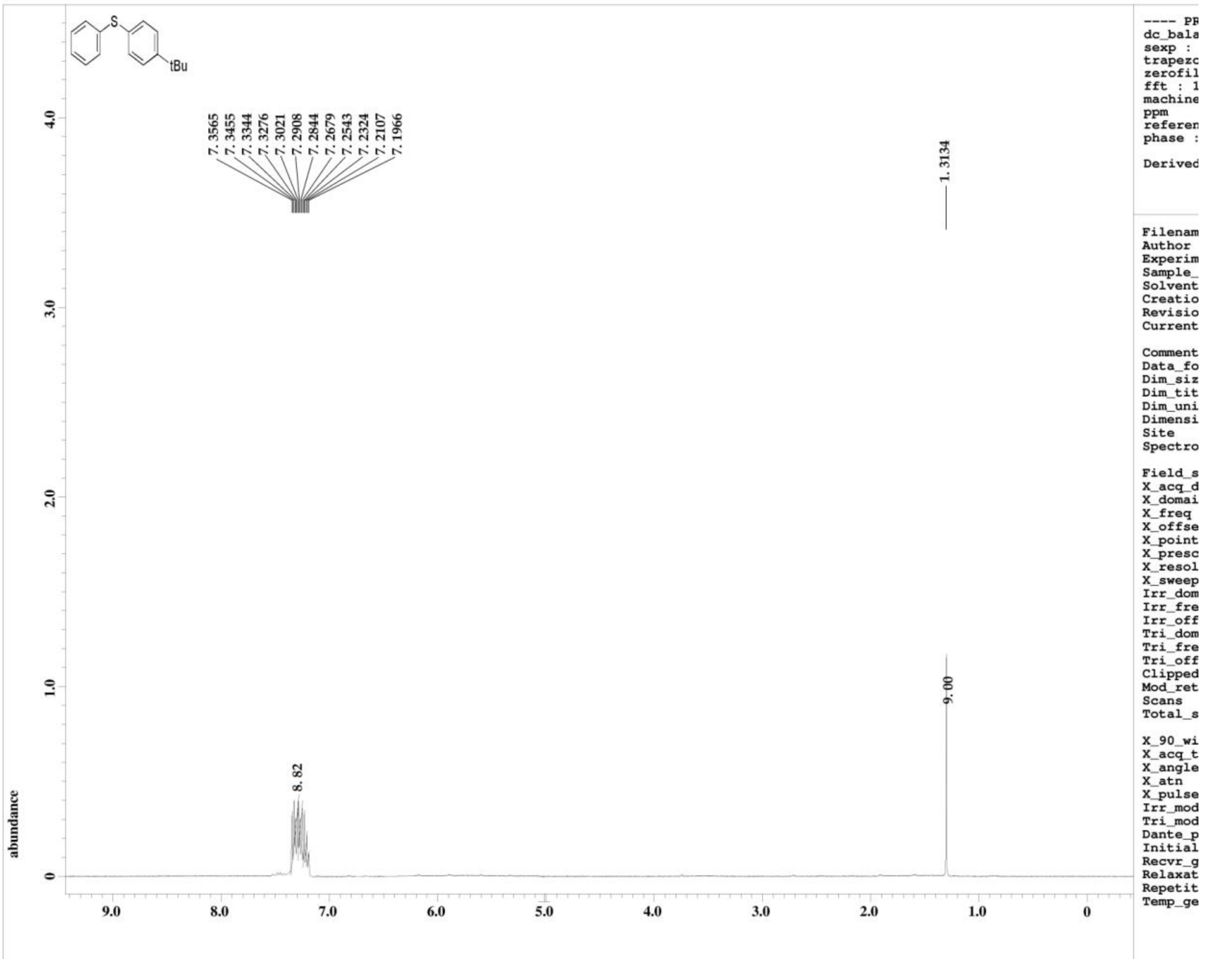
tBu
S
7.3565
7.3455
7.3344
7.3276
7.3021
7.2908
7.2844
7.2679
7.2543
7.2324
7.2107
7.1966
1.3134
8.82
9.00
abundance
9.0
8.0
7.0
6.0
5.0
4.0
3.0
2.0
1.0
0

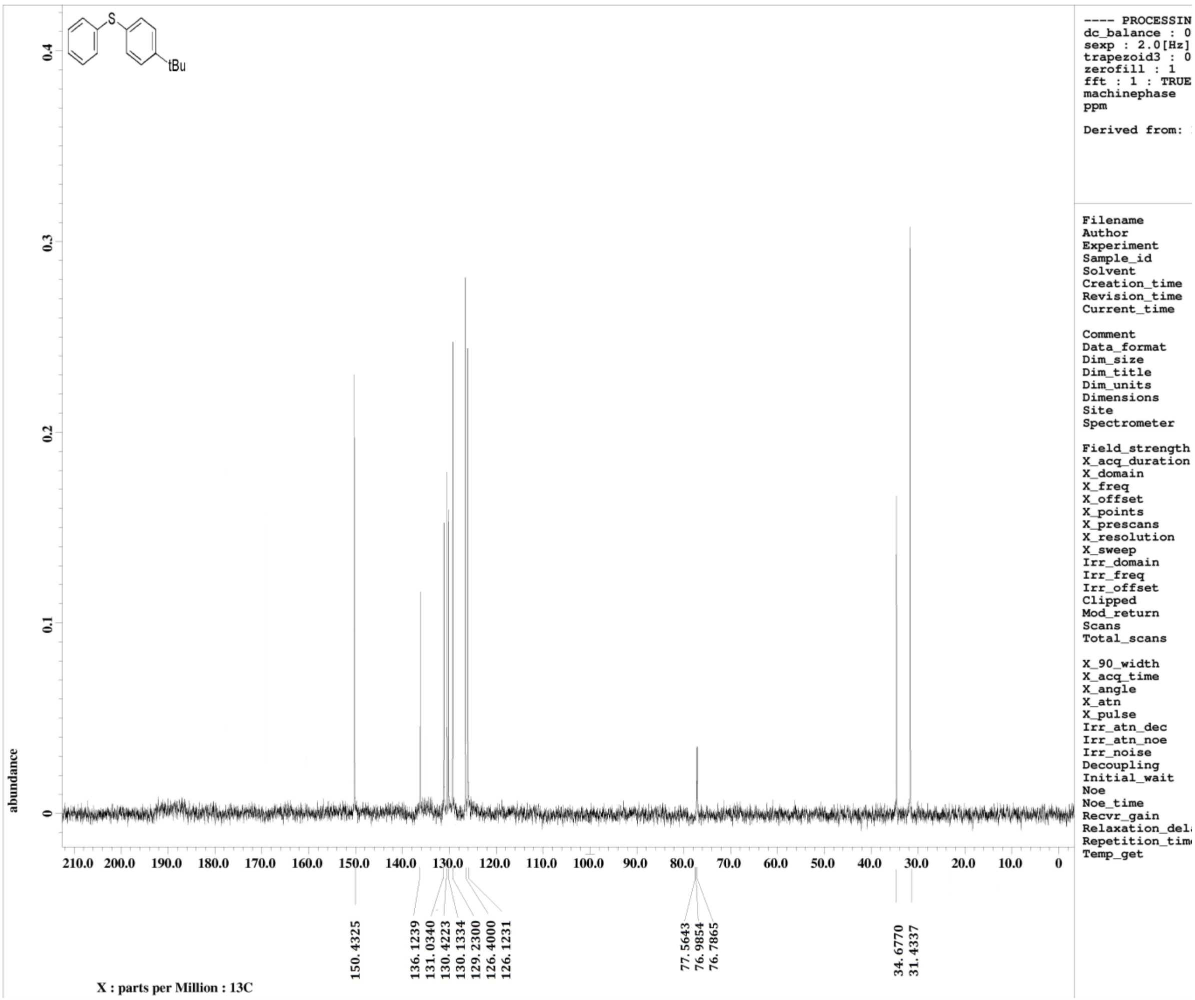

tBu
S
abundance
X : parts per Million : 13C
150.4325
136.1239
131.0340
130.4223
130.1334
129.2300
126.4000
126.1231
77.5643
76.9854
76.7865
34.6770
31.4337

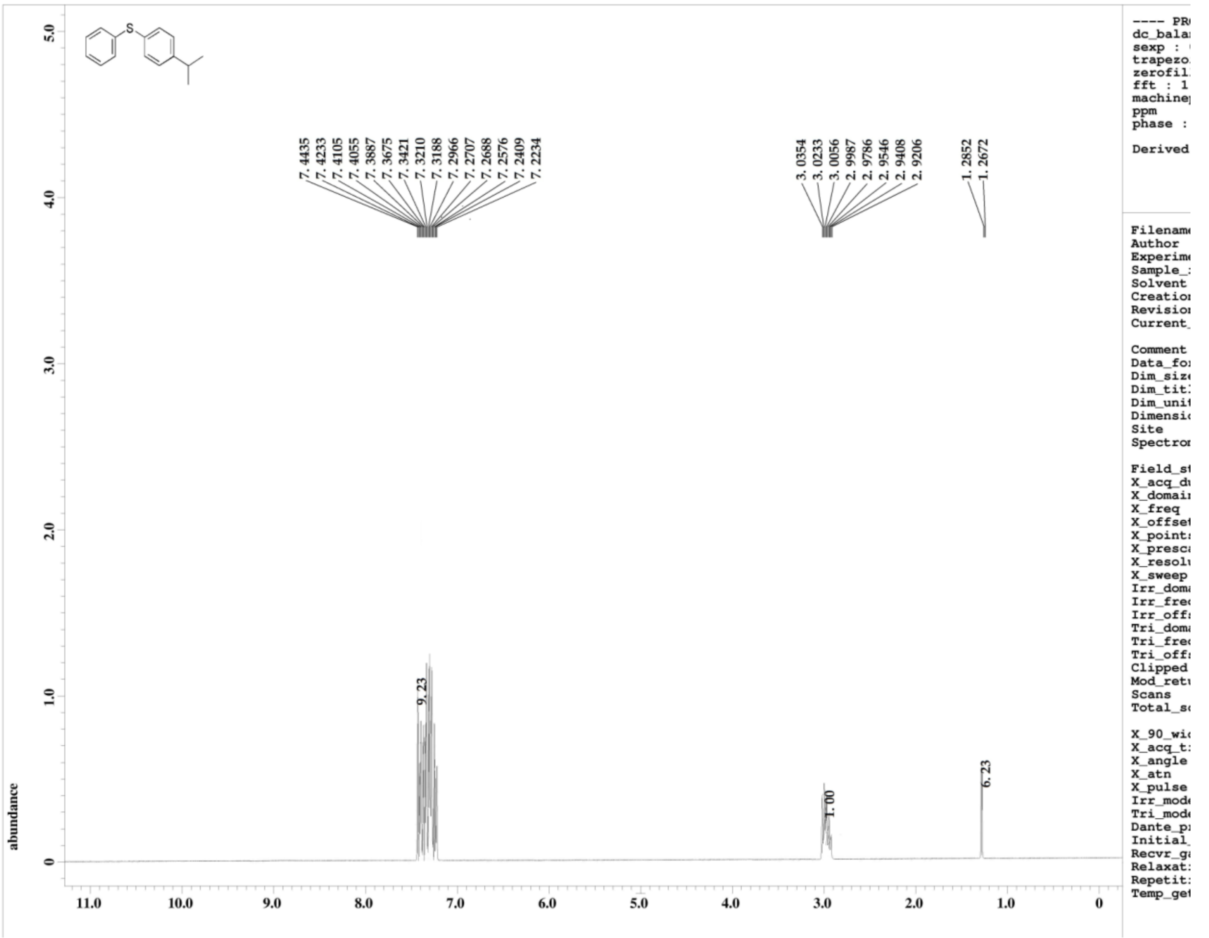

7.4435
7.4233
7.4105
7.4055
7.3887
7.3675
7.3421
7.3210
7.3188
7.2966
7.2707
7.2688
7.2576
7.2409
7.2234
3.0354
3.0233
3.0056
2.9987
2.9786
2.9546
2.9408
2.9206
1.2852
1.2672
9.23
1.00
6.23
abundance

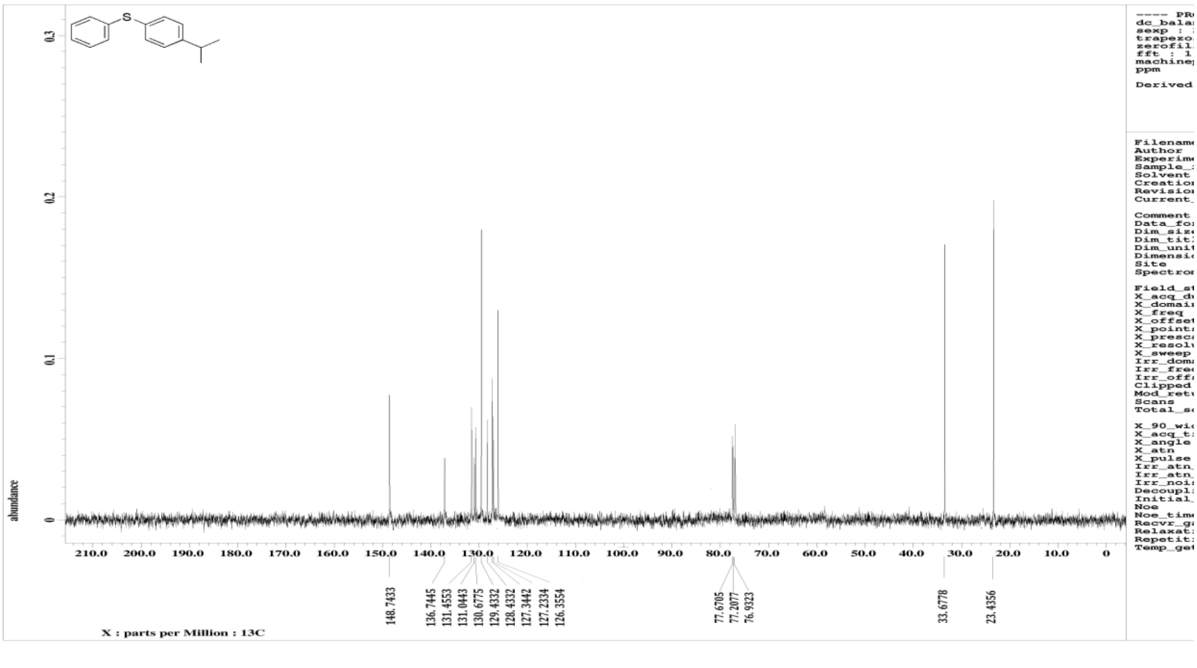

148.7433
136.7445
131.4553
131.0443
130.6775
129.4332
128.4332
127.3442
127.2334
126.3554
77.6705
77.2077
76.9323
33.6778
23.4356
abundance
X : parts per Million : 13C

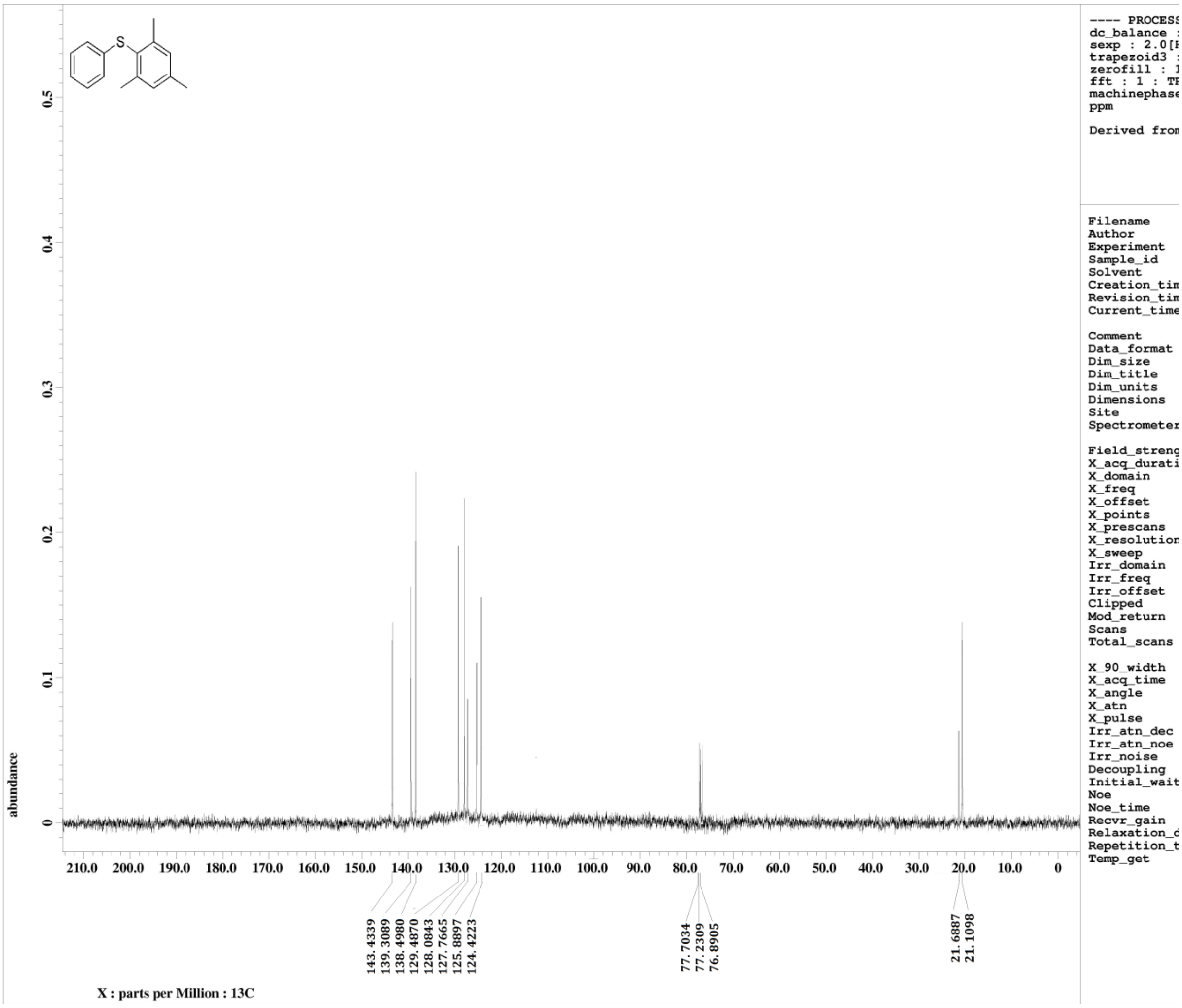
---- PROCESS
dc_balance :
sexp : 2.0[
trapezoid3 :
zerofill : 1
fft : 1 : T
machinephase
ppm
Derived from
Filename
Author
Experiment
Sample_id
Solvent
Creation_tim
Revision_tim
Current_time
Comment
Data_format
Dim_size
Dim_title
Dim_units
Dimensions
Site
Spectrometer
Field_streng
X_acq_durati
X_domain
X_freq
X_offset
X_points
X_prescans
X_resolution
X_sweep
Irr_domain
Irr_freq
Irr_offset
Clipped
Mod_return
Scans
Total_scans
X_90_width
X_acq_time
X_angle
X_atn
X_pulse
Irr_atn_dec
Irr_atn_noe
Irr_noise
Decoupling
Initial_wait
Noe
Noe_time
Recvr_gain
Relaxation_d
Repetition_t
Temp_get
abundance
143.4339
139.3089
138.4980
129.4870
128.0843
127.7665
125.8897
124.4223
77.7034
77.2309
76.8905
21.6887
21.1098
X : parts per Million : 13C

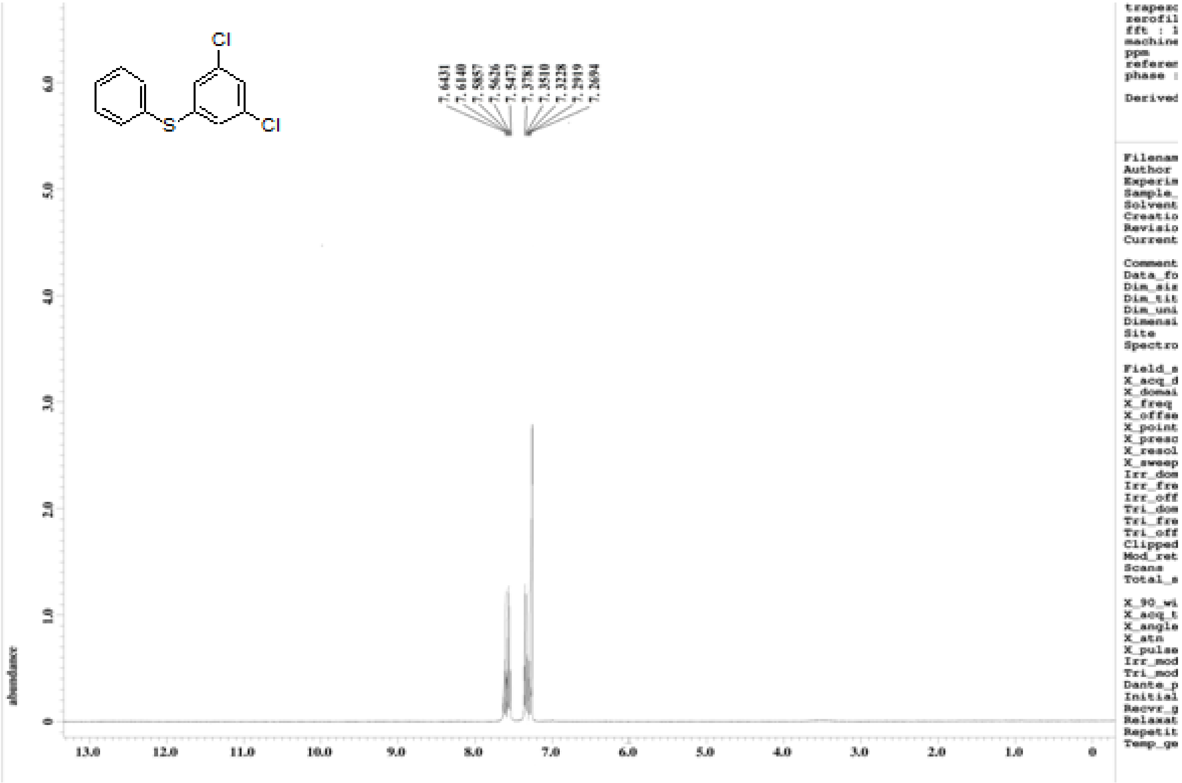
Cl
S
Cl
7.6431
7.6140
7.5857
7.5626
7.5473
7.3781
7.3510
7.3228
7.2919
7.2694
abundance

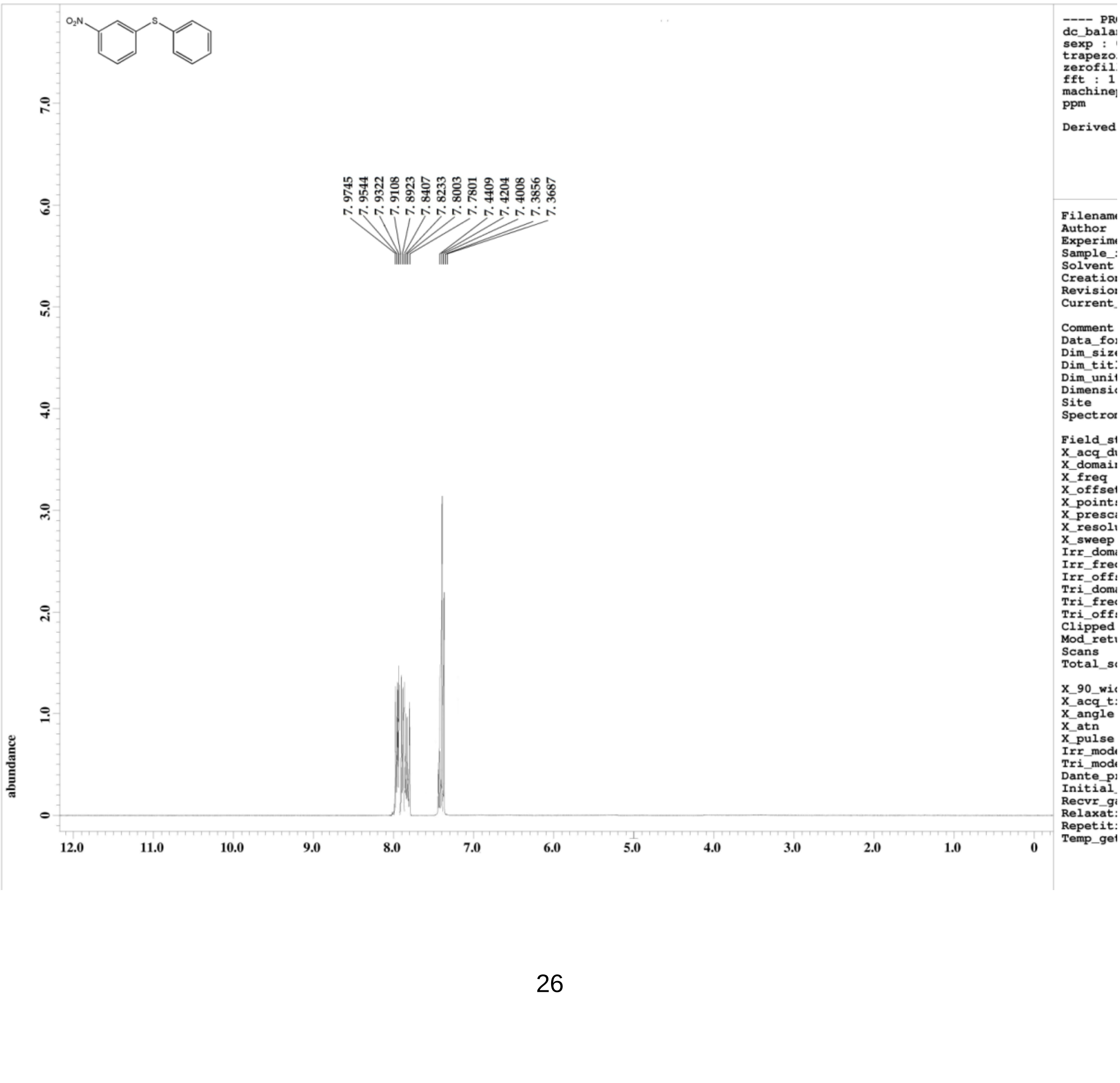
O2N
S
7.9745
7.9544
7.9322
7.9108
7.8923
7.8407
7.8233
7.8003
7.7801
7.4409
7.4204
7.4008
7.3856
7.3687
abundance

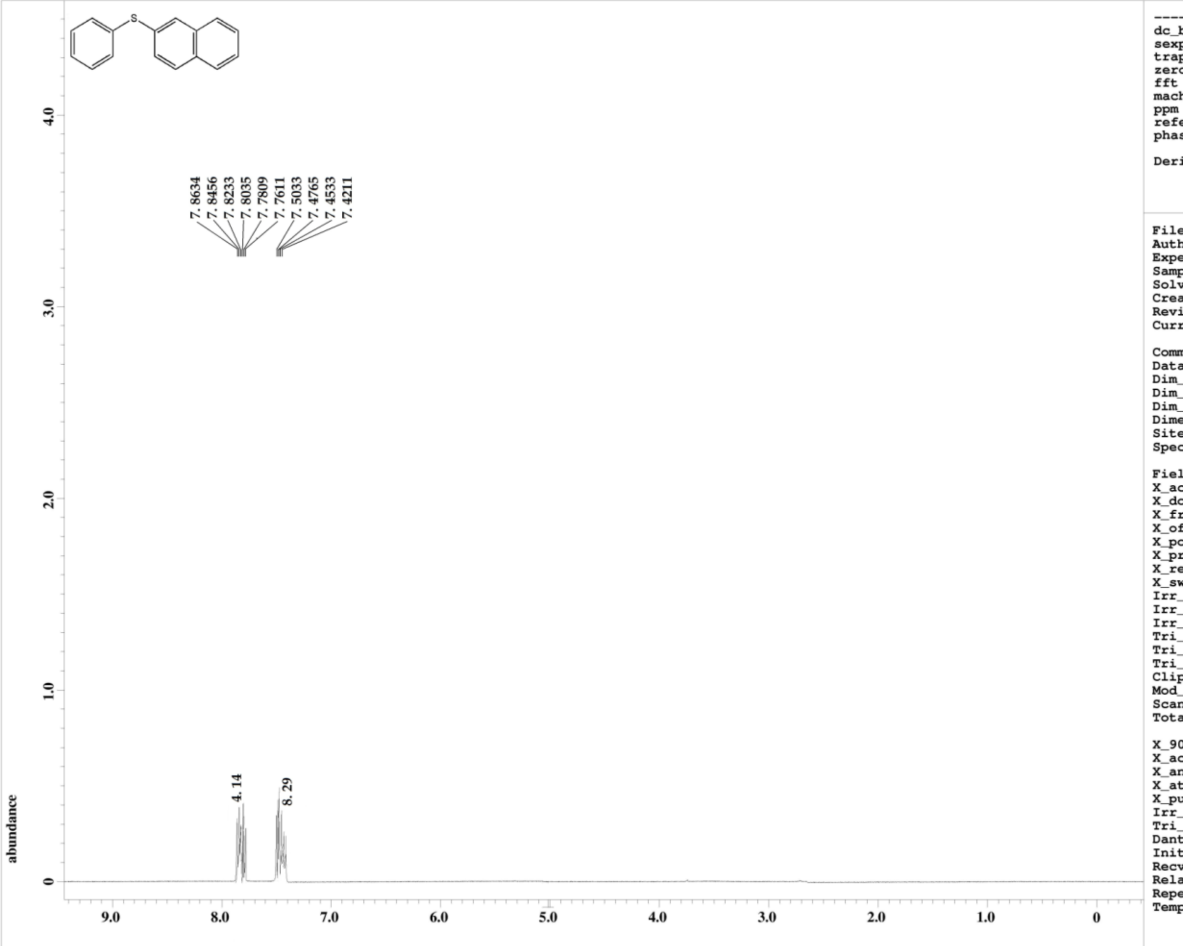
S
7.8634
7.8456
7.8233
7.8035
7.7809
7.7611
7.5033
7.4765
7.4533
7.4211
4.14
8.29
abundance
9.0
8.0
7.0
6.0
5.0
4.0
3.0
2.0
1.0
0

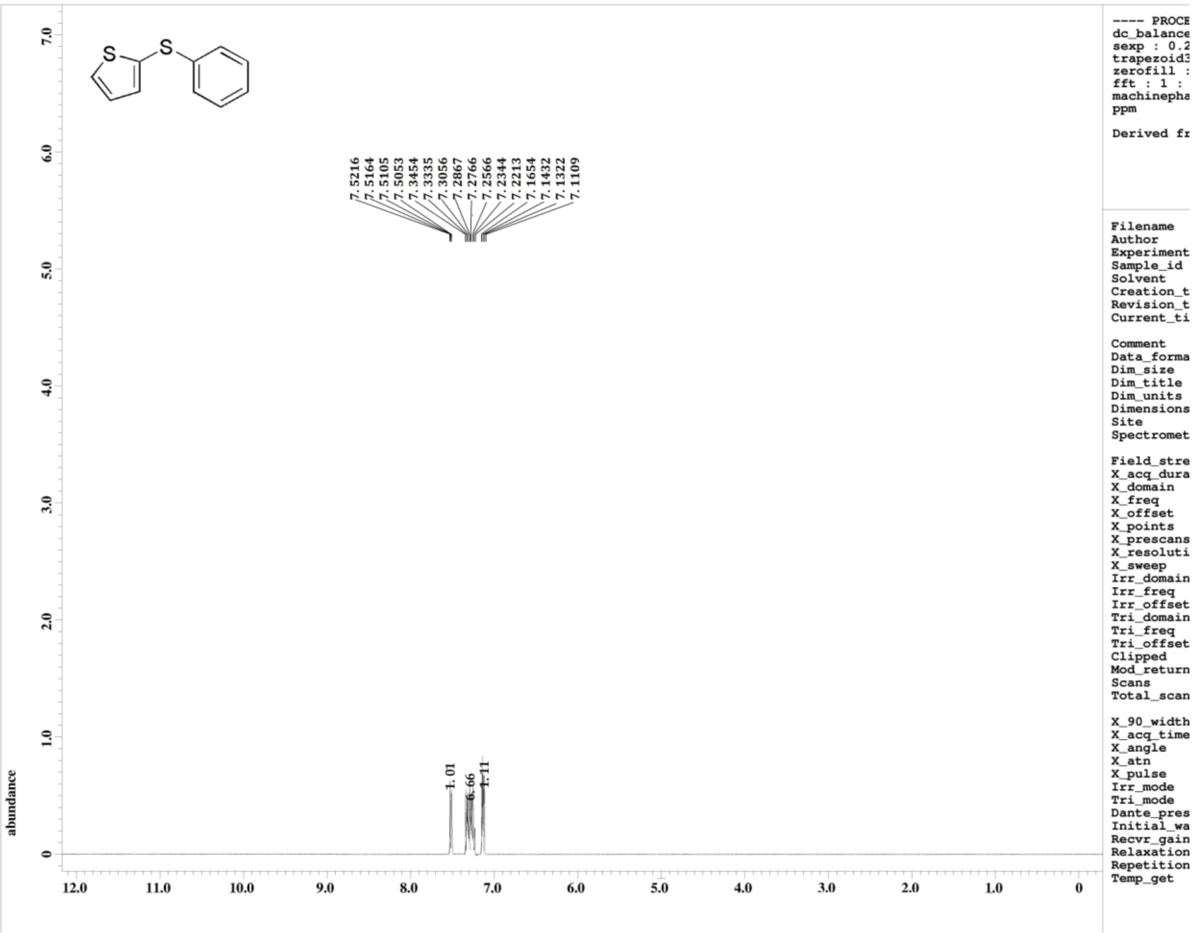

7.5216
7.5164
7.5105
7.5053
7.3454
7.3335
7.3056
7.2867
7.2766
7.2566
7.2344
7.2213
7.1654
7.1432
7.1322
7.1109
1.01
6.66
1.11
abundance
12.0
11.0
10.0
9.0
8.0
7.0
6.0
5.0
4.0
3.0
2.0
1.0
0
---- PROCE
dc_balance
sexp : 0.2
trapezoid3
zerofill :
fft : 1 :
machinepha
ppm
Derived fr
Filename
Author
Experiment
Sample_id
Solvent
Creation_t
Revision_t
Current_ti
Comment
Data_forma
Dim_size
Dim_title
Dim_units
Dimensions
Site
Spectromet
Field_stre
X_acq_dura
X_domain
X_freq
X_offset
X_points
X_prescans
X_resoluti
X_sweep
Irr_domain
Irr_freq
Irr_offset
Tri_domain
Tri_freq
Tri_offset
Clipped
Mod_return
Scans
Total_scan
X_90_width
X_acq_time
X_angle
X_atn
X_pulse
Irr_mode
Tri_mode
Dante_pres
Initial_wa
Recvr_gain
Relaxation
Repetition
Temp_get

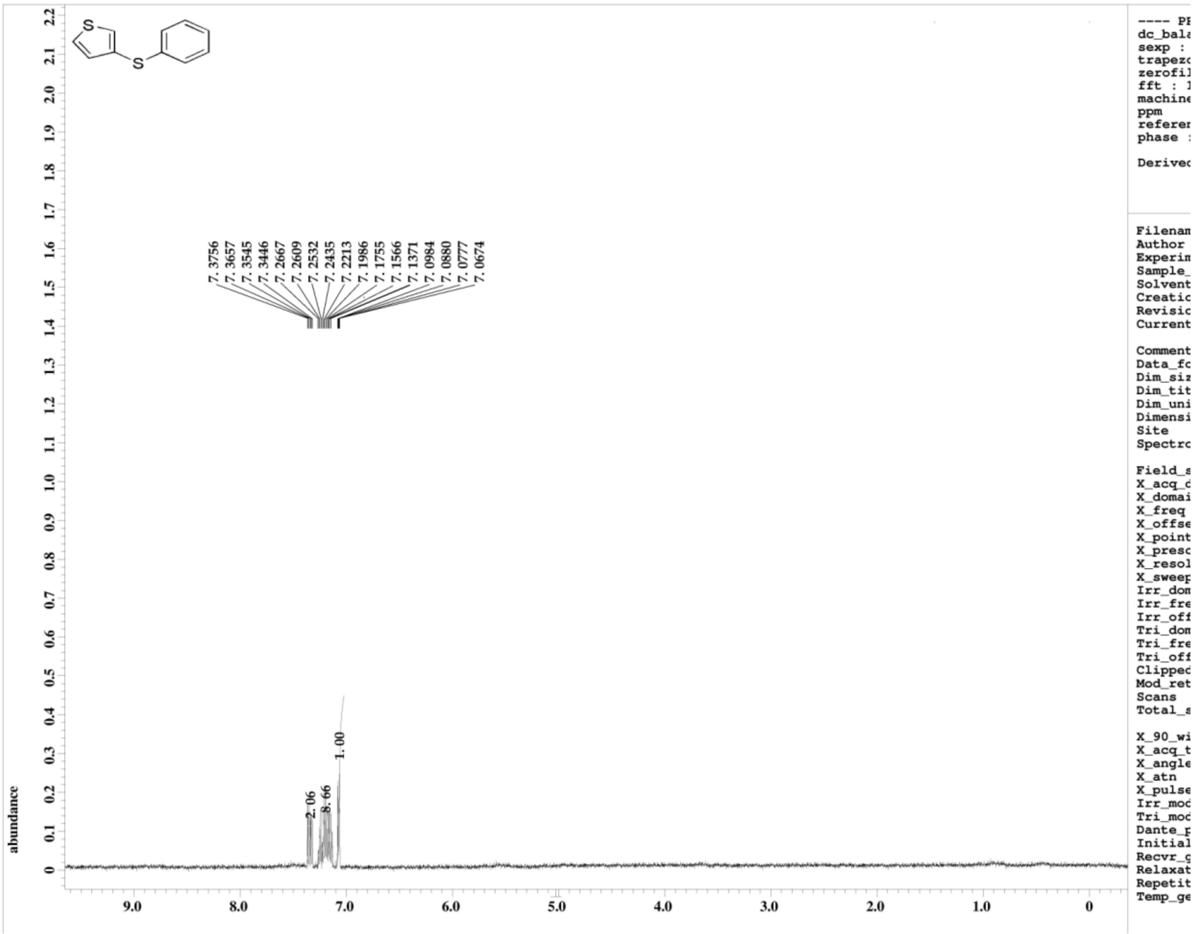
S
S
7.3756
7.3657
7.3545
7.3446
7.2667
7.2609
7.2532
7.2435
7.2213
7.1986
7.1755
7.1566
7.1371
7.0984
7.0880
7.0777
7.0674
2.06
8.66
1.00
abundance
9.0
8.0
7.0
6.0
5.0
4.0
3.0
2.0
1.0
0

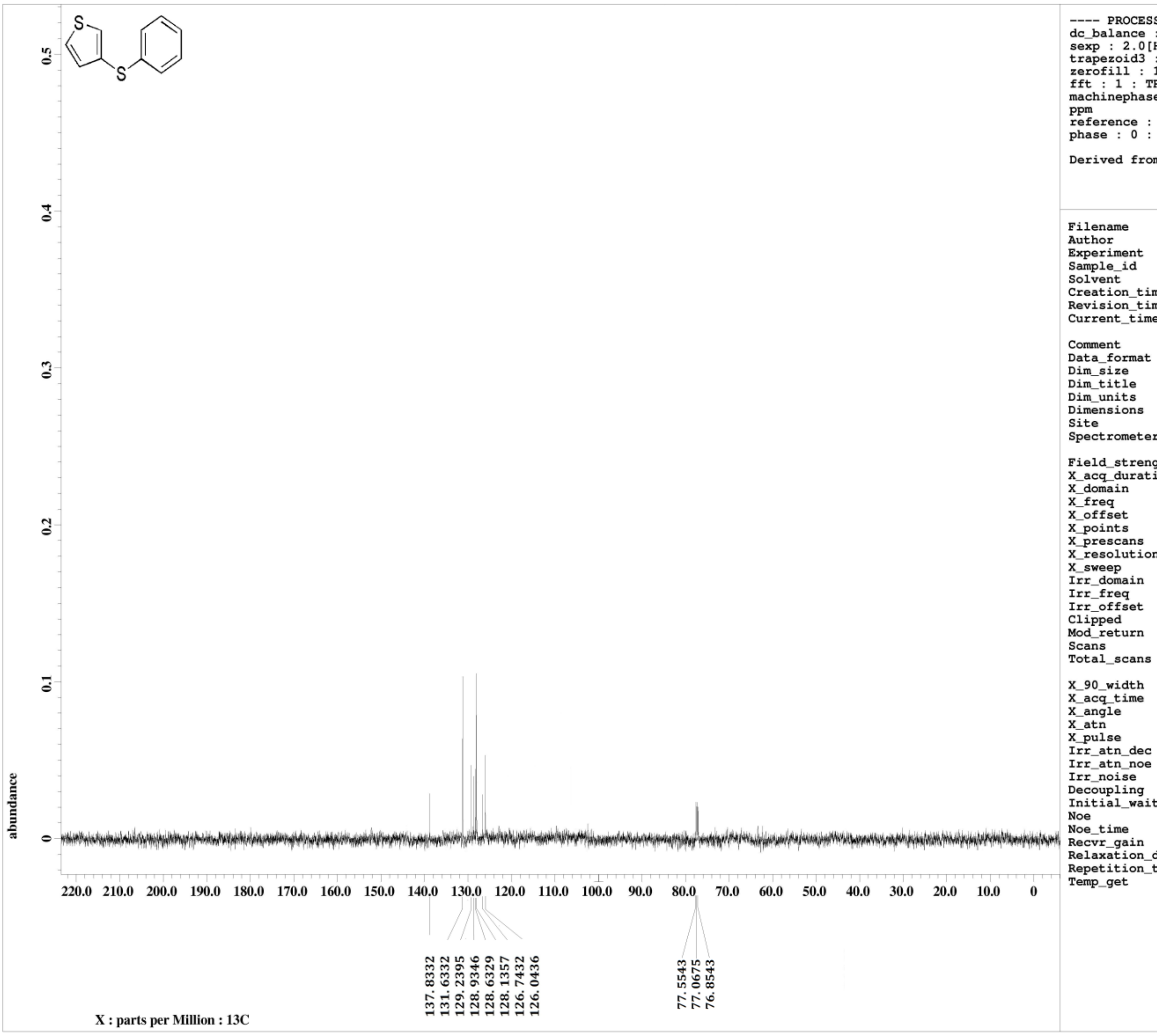

abundance
X : parts per Million : 13C
137.8332
131.6332
129.2395
128.9346
128.6329
128.1357
126.7432
126.0436
77.5543
77.0675
76.8543
